# Heat-inactivated *Bifidobacterium adolescentis* ameliorates colon senescence through Paneth-like-cell-mediated stem cell activation

**DOI:** 10.1038/s41467-023-41827-0

**Published:** 2023-09-30

**Authors:** Yadong Qi, Jiamin He, Yawen Zhang, Qiwei Ge, Qiwen Wang, Luyi Chen, Jilei Xu, Lan Wang, Xueqin Chen, Dingjiacheng Jia, Yifeng Lin, Chaochao Xu, Ying Zhang, Tongyao Hou, Jianmin Si, Shujie Chen, Liangjing Wang

**Affiliations:** 1 Department of Gastroenterology, Sir Run Run Shaw Hospital, Zhejiang University School of Medicine, Hangzhou, Zhejiang China; 2 Institute of Gastroenterology, Zhejiang University, Hangzhou, Zhejiang China; 3 Prevention and Treatment Research Center for Senescent Disease, Zhejiang University School of Medicine, Hangzhou, Zhejiang China; 4 Department of Gastroenterology, Second Affiliated Hospital of Zhejiang University School of Medicine, Hangzhou, Zhejiang China; 5 Department of General Practice, Sir Run Run Shaw Hospital, Zhejiang University School of Medicine, Hangzhou, Zhejiang China

**Keywords:** Microbiology, Gastroenterology

## Abstract

Declined numbers and weakened functions of intestinal stem cells (ISCs) impair the integrity of the intestinal epithelium during aging. However, the impact of intestinal microbiota on ISCs in this process is unclear. Here, using premature aging mice (telomerase RNA component knockout, *Terc*^*−/−*^), natural aging mice, and in vitro colonoid models, we explore how heat-inactivated *Bifidobacterium adolescentis* (*B. adolescentis*) affects colon senescence. We find that *B. adolescentis* could mitigate colonic senescence-related changes by enhancing intestinal integrity and stimulating the regeneration of Lgr5^+^ ISCs via Wnt/β-catenin signaling. Furthermore, we uncover the involvement of Paneth-like cells (PLCs) within the colonic stem-cell-supporting niche in the *B. adolescentis-*induced ISC regeneration. In addition, we identify soluble polysaccharides (SPS) as potential effective components of *B. adolescentis*. Overall, our findings reveal the role of heat-inactivated *B. adolescentis* in maintaining the ISCs regeneration and intestinal barrier, and propose a microbiota target for ameliorating colon senescence.

## Abstract (web summary)

Here, Wang *et al*. show that heat-inactivated *Bifidobacterium adolescentis* mediates maintenance of host intestinal barrier through intestinal stem cells activation, which can potentially have translational implications for alleviation of colon senescence and related diseases.

## Introduction

Intestinal epithelium is one of the most rapidly turnover tissues in the human body, which keeps the host from various harmful environmental factors and pathogens initially^1^. Intestinal homeostasis has a profound impact on multiple systems. The self-renewal of intestinal stem cells (ISCs) is an important process to maintain intestinal integrity^2^. Previous studies have demonstrated that intestine senescence is accompanied by dysfunction and exhaustion of the ISCs^3–6^, which further increases the incidence of age-related intestinal diseases, such as chronic constipation, colonic diverticular disease, malnutrition, and colorectal cancer^7^. Therefore, exploring a better strategy to promote ISCs regeneration may offer a bright view to rejuvenate the intestine senescence^8^.

Growing evidence has suggested that gut microbiota undergo massive changes across the lifetime. Imbalanced shifts in microbial composition are associated with age^9^. This age-related microbial dysbiosis further triggers a chain of inflammatory events, causes gut leakage, and affects the overall health of the host^8,10^. A recent study has provided evidence that fecal microbiota transplantation has the potential to mitigate the accelerated-aging phenotype and significantly prolong the lifespan in progeroid mice^11^. This offers a chance to slow or even reverse age-related changes and diseases through microbial interventions, such as probiotics, synbiotics, and microbiota-targeted diet changes. Our previous studies also demonstrated that heat-inactivated *B. adolescentis*, one commensal bacterium, dominated in the young adult intestine, could improve the healthspan and lifespan in multiple species^12^.

In this study, we uncovered the protective role of heat-inactivated *B. adolescentis* in the colon senescence and revealed that the regeneration of the ISCs was mediated by the Wnt/β-catenin signaling and supported by Paneth-like cells. We identified the soluble polysaccharides (SPS) derived from heat-inactivated *B. adolescentis* as a potential effective component.

## Results

### The stemness and integrity of the senescent colon were related to the abundance of *B*. *adolescentis*

**Fig. 1:**
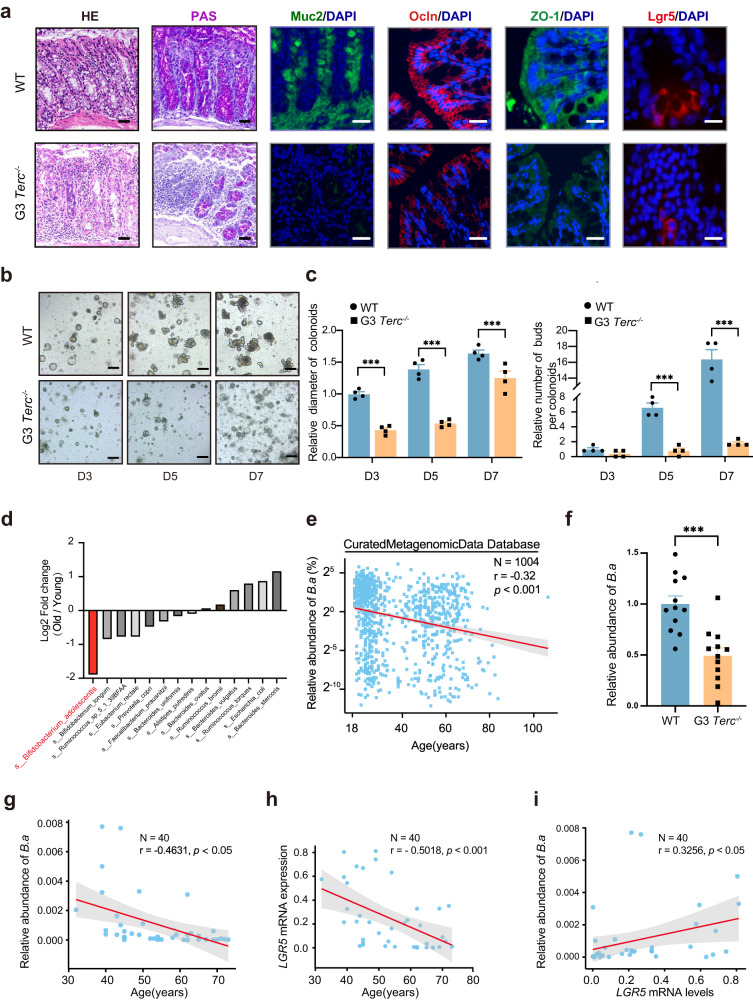
The association between senescent colon characterized by impaired intestinal integrity and reduced stemness with *B. adolescentis* abundance. **a** Representative images of H&E staining, PAS staining, immunofluorescence staining of Muc2 (green), ZO-1 and Ocln and Lgr5 in the colon of WT and G3 *Terc*^*-/-*^ mice. Scale bar, 50 μm. *n* = 6 animals *per* group. **b** Representative images (growing on day 3, 5 and 7) of colonoids derived from WT and G3 *Terc*^*-/-*^ mice, Scale bar, 100 μm. Representative pictures are shown, *n* = 4 independent experiments. **c** Comparison of size and de novo buds’ number in colonoids derived from WT and G3 *Terc*^*-/-*^ mice. *n* = 4 independent experiments. Comparisons were performed by Two-way ANOVA analysis followed by Bonferroni’s multiple comparisons test. **d** The fold change differences (Young/Old group) of the top shared microbiota in the CuratedMetagenomicData. **e** Correlation analysis between the relative abundance of *B. adolescentis* and age in CuratedMetagenomicData Database (*n* = 1004, Spearman *r* = −0.32, *p* < 0.001). Comparisons were performed by Spearman’s correlation analysis. **f** Comparison of fecal *B. adolescentis* abundance between WT and G3 *Terc*^*-/-*^ mice (*n* = 12 animals *per* group). Data were represented as mean ± SEM. Comparisons were performed by unpaired, two-tailed *t* test. **g** Correlation analysis between the abundance of *B. adolescentis* and different ages in human colonic tissues (*n* = 40, Pearson *r* = −0.4631, *p* < 0.05). Comparisons were performed by Pearson’s correlation analysis. **h** Correlation analysis between the expression of the *LGR5* gene in human colonic tissues and different ages (*n* = 40, Pearson *r* = −0.5018, *p* < 0.001). Comparisons were performed by Pearson’s correlation analysis. **i** Correlation analysis between the abundance of *B. adolescentis* and the *LGR5* gene in human colonic tissues (*n* = 40, Pearson *r* = 0.3256, *p* < 0.05). Comparisons were performed by Pearson’s correlation analysis. ***p < 0.001, Source data and exact *p* value are provided as a Source data file.

The age-related phenotypes of progeroid mice (G3 *Terc*^*-/-*^ mice) including weight loss, overall frailty, bone mass, and neuron changes have been well characterized^12^. We further focused on the age-related morphological changes in the colon (Fig. 1a) and found that the mucosa of the colon in 7-month-old G3 *Terc*^*-/-*^ mice was thinner than the wild type (WT) controls. G3 *Terc*^*-/-*^ mice showed impaired intestinal barrier presenting as less secretory goblet cells and lower expression of intestinal integrity-related genes (ZO-1 and *Ocln*). Meanwhile, the number of Lgr5^+^ ISCs significantly decreased in the G3 *Terc*^*-/-*^ mice. The organoid-forming capacity of ISC-containing colonic crypts was reported as an in vitro assay for intestinal regenerative potential. We observed a significant reduction in the organoid-forming capacity of the crypts in G3 *Terc*^*-/-*^ mice, manifesting as fewer colonoids, smaller size, and fewer de novo buds (Fig. 1b, c).

In light of this, we further explored GSE94515 database and found that the relative expression of two typical senescent-associated genes (*p16* and *p21*) in the mouse colon were significantly increased with aging, while *Lgr5* and *Ocln* expression decreased accordingly (Supplementary Fig. 1a). FITC-dextran concentration levels in peripheral blood and the LPS activity in the fecal and serum samples were also significantly increased in aged mice, which indicated the declined intestinal integrity (Supplementary Fig. 1b). We further confirmed that the gene expression of *p16* and *p21* elevated in the aged human colon, which was accompanied by reduced gene expression of *LGR5* and *OCLN* from the healthy human tissue database (GTEx) (Supplementary Fig. 1c). There was a positive correlation between the expression of *OCLN* and *LGR5* genes in human colon (*r* = 0.78, *p* < 0.001, Supplementary Fig. 1d). These results indicated that senescent colon had impaired epithelial integrity with the increased intestinal permeability and reduced stemness.

It is also well recognized that aging is associated with gut microbiome changes^13^. Our previous 16S rRNA sequencing results from a healthy human cohort showed that fecal *B. adolescentis* decreased with aging^12^. We further ranked the intestinal bacteria abundance in healthy young or old human cohorts shared in the public metagenomic database (CuratedMetagenomicData). As expected, *B. adolescentis* showed the most significant difference between the young and the old populations (Fig. 1d and Supplementary Fig. 1e), and the relative abundance of *B. adolescentis* was negatively correlative with age (*r* = − 0.32, *p* < 0.001, Fig. 1e and Supplementary Fig. 1f). We found that the relative abundance of *B. adolescentis* in the feces of G3 *Terc*^*-/-*^ mice was lower than that in WT mice (Fig. 1f).

To better explore the relationship between *B. adolescentis* and intestinal stemness in the senescent colon, we collected colon biopsies from healthy human donors to measure the *LGR5* level and *B. adolescentis* load. The expression of *LGR5* and abundance of *B. adolescentis* both negatively correlated with the age (Fig. 1g, h), and the low abundance of *B. adolescentis* was positively associated with the reduced level of *LGR5* (*r* = 0.3256, *p* < 0.05, Fig. 1i). Additionally, the altered *B. adolescentis* was accompanied with complementary alteration in other species in the complex microbiome, such as *Bifidobacterium pseudocatenulatum* (Supplementary Fig. 1g).

### Heat-inactivated *B. adolescentis* alleviated colon senescence phenotypes by intestinal barrier improvement and ISCs restoration

**Fig. 2:**
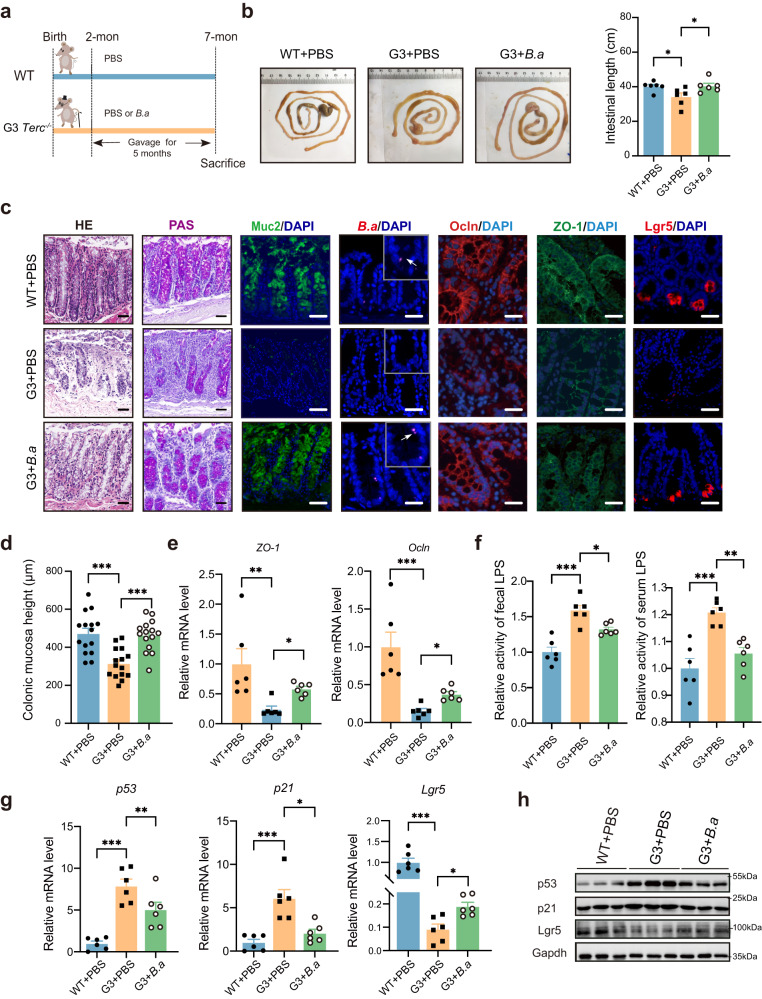
Heated-inactivated *B. adolescentis* alleviated colon senescence phenotype by improving the intestinal barrier and restoring ISCs in G3 *Terc*^-/-^ mice. **a** The schematic diagram of the WT and G3 *Terc*^-/-^ mice experimental procedure. **b** Representative image of gross morphology and length analysis of the mouse colon. *n* = 6 animals *per* group, the error bars indicate the mean ± SEM. Comparisons were performed by One-way ANOVA analysis followed by Dunnett’s multiple comparisons test. **c** Representative image of H&E staining and PAS staining, immunofluorescence image of Muc2, ZO-1 and Ocln and Lgr5, FISH probe of *B. adolescentis* in the colon from WT + PBS, G3 *Terc*^*-/-*^ + PBS and G3 *Terc*^-/-^ + *B.a* mice. Scale bar, 50 μm, *n* = 6 animals *per* group. **d** The mucosal height was measured. *n* = 15 random fields *per* group. Data were represented as mean ± SEM. Comparisons were performed One-way ANOVA analysis followed by Tukey’s multiple comparisons test. **e** Relative mRNA levels of ZO-1 and *Ocln* genes in WT + PBS, G3 *Terc*^*-/-*^ + PBS and G3 *Terc*^*-/-*^ + *B.a* mice. *n* = 6 animals *per* group. Data were represented as mean ± SEM. Comparisons were performed by Kruskal–Wallis test followed by Two-stage linear step-up procedure of Benjamini, Krieger and Yekutieli. **f** Relative LPS levels in the fecal and serum samples in WT + PBS, G3 Terc^-/-^ + PBS and G3 Terc^-/-^ + *B.a* mice. *n* = 6 animals *per* group. Data were represented as mean ± SEM. Comparisons were performed by One-way ANOVA analysis followed by Tukey’s multiple comparisons test. **g** Relative mRNA levels of *p53*, *p21* and *Lgr5* gene in WT + PBS, G3 *Terc*^-/-^ + PBS and G3 *Terc*^-/-^ + *B.a* mice. *n* = 6 animals *per* group. Data were represented as mean ± SEM. Comparisons in *p53*, *p21* were performed by One-way ANOVA analysis followed by Tukey’s multiple comparisons test and in *Lgr5* were performed by Kruskal–Wallis test followed by two-stage linear step-up procedure of Benjamini, Krieger and Yekutieli. **h** The protein level of p53, p21 and Lgr5 were detected by immunoblot from WT + PBS, G3 *Terc*^-/-^ + PBS and G3 *Terc*^-/-^ + *B.a* mice. *p < 0.05, **p < 0.01, ***p < 0.001. Source data and exact *p* value are provided as a Source data file.

To identify the anti-aging effect of heat-inactivated *B. adolescentis* in the colon, 2-month-old G3 *Terc*^-/-^ mice were intragastrically administrated with *B. adolescentis* ATCC 15703 (pretreated with heat-inactivation as previously described^12^, and all the bacteria used below were heat-inactivated) or phosphate-buffered saline (PBS) for 5 months, whereas the age and gender-matched wild-type (WT) *Terc*^+/+^ mice were administrated with PBS as control (Fig. 2a). We found heat-inactivated *B. adolescentis* supplementation prolonged the shortened intestine of G3 *Terc*^-/-^ mice (Fig. 2b). Histological analysis results demonstrated that crypts-located *B. adolescentis* increased the thickness of colonic mucosa, enriched the number of secretory goblet cells and Lgr5^+^ ISCs, and improved intestinal barrier integrity as evidenced by the upregulation of transcript levels of tight junction proteins (ZO-1 and Ocln) and the reduction of lipopolysaccharide (LPS) levels in both fecal and serum samples (Fig. 2c–f).

To further assess the effect of *B. adolescentis* on colon senescence, we compared the mRNA and protein expression levels of *p21*, *p53* and *Lgr5*. The results showed that *B. adolescentis* could significantly suppress the expression of *p21* and *p53* in G3 *Terc*^-/-^ mice colon, and restore the expression of *Lgr5* (Fig. 2g, h). In addition, we analyzed the fecal microbial changes and observed that the α-diversity (indicated by the Shannon index) and the β-diversity were significantly different between *B. adolescentis*-treated and control groups. Proportions of the Firmicutes and the Bacteroidetes (F/B) were increased after administration with *B. adolescentis* (Supplementary Fig. 2).

To further validate the impact of heat-inactivated *B. adolescentis* on colon senescence, we employed the natural aging mice model as well (Supplementary Fig. 3a). The outcomes obtained from this model exhibited similarities to those observed in the G3 *Terc*^-/-^ mice (Supplementary Fig. 3b–h). Collectively, these results indicated that heat-inactivated *B. adolescentis* could alleviate colon senescence by improving intestinal barrier function and restoring ISCs.

### Heat-inactivated *B. adolescentis* intervention promoted the regenerative potential of ISCs

**Fig. 3:**
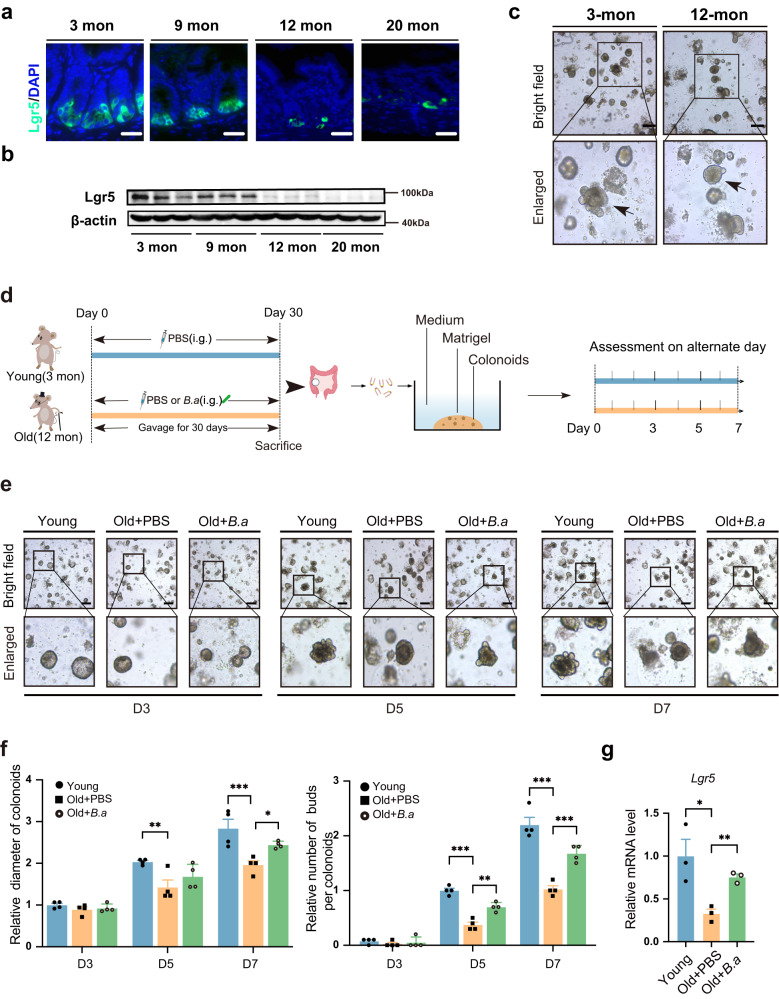
Heated-inactivated *B. adolescentis* supplementation improved colonoids performance in vivo. **a**, **b** The expression of the Lgr5 gene was detected in mice colon tissue from different age groups by immunofluorescence and western blot, *n* = 3 animals *per* age group. Scale bar, 50 μm. **c** Representative images of colonoids derived from 3-month-old mice and 12-month-old mice with *n* = 3 independent experiments with similar results. Arrows indicate crypt domains. Scale bar, 100 μm. **d** The schematic diagram of the experimental procedure in the young (3-month-old mice) and old (12-month-old mice) groups. **e** Representative images are shown from one out of 4 independent experiments with similar results in the organoid-forming capacity of crypts from Young, Old+PBS and Old+*B.a* mice group. Scale bar, 100 μm. **f** Relative size of colonoids quantified on days 3, 5 and 7 and represented relative to the Young group. *n* = 4 independent experiments *per* group) on days 3, 5 and 7. Data were represented as mean ± SEM. Comparisons were performed by Two-way ANOVA analysis followed by Tukey’s multiple comparisons test. **g** Relative mRNA levels of the Lgr5 gene in Young, Old+PBS and Old+*B.a* mice group, *n* = 3 individual experiments. Data were represented as mean ± SEM. Comparisons were performed by unpaired, two-tailed *t* test. *p < 0.05, **p < 0.01, ***p < 0.001. Source data and exact *p* value are provided as a Source data file.

Our results demonstrated a decrease in the protein expression of Lgr5 at the age of 12-month-old compared to 3-month-old (Fig. 3a, b). This observation was further confirmed by colonoid formation assays (Fig. 3c). These findings are consistent with previous reports (GSE186811) analyzing Lgr5^hi^ cells from 12-month-old mice with bulk RNAseq which showed >95% of stem cell signature genes were down-regulated at 12 months when compared to 3 months^14,15^, which indicated that the stem cells damage appeared as early as middle age (12-month-old mouse)^16^.

We then conducted a 1-month heat-inactivated *B. adolescentis* administration (10^9^ CFU) in young (3 months) and old (12 months) mice. Then colonic crypts were isolated and cultured in Matrigel to assess the regenerative potential^17^. We captured and calculated the growth parameters of colonoids on days 3, 5 and 7, respectively (Fig. 3d). Morphologically, the diameter of colonoids was larger and the proportion of budding colonoids became higher at day 5 and day 7 (Fig. 3e, f). The results demonstrated that *B. adolescentis* could improve the regenerative potential of ISCs, which was verified by the *Lgr5* mRNA levels (Fig. 3g).

**Fig. 4:**
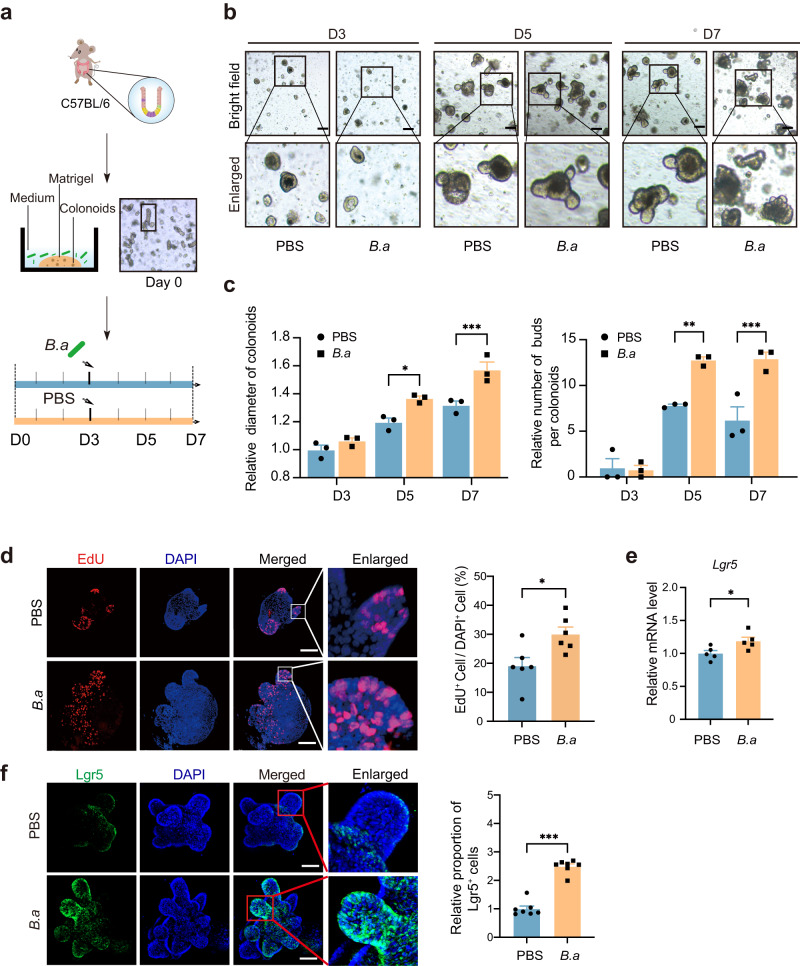
Heated-inactivated *B. adolescentis* increased the proliferation of colonoids in vitro. **a** The schematic diagram of the experimental procedure in mice colonoids. **b** Representative images with 3 independent experiments in the organoid-forming capacity of crypts from PBS or *B.a* treated group. Scale bar, 100 μm. **c** The relative size of colonoids was quantified on days 3, 5 and 7 and represented relative to PBS treated group (*n* = 3 independent experiments *per* group). Data were represented as mean ± SEM. Comparisons were performed by Two-way ANOVA analysis followed by Bonferroni’s multiple comparisons test. **d** Representative images of colonoids staining with DAPI (blue) and EdU (red); Scale bar, 50 μm. The mean density of EdU-positive cells in each group was calculated. *n* = 6 random field of view *per* group in three independent experiments. Data were presented as the mean ± SEM. Comparisons were performed by unpaired, two-tailed *t* test **e** Relative expression of Lgr5 gene in PBS and *B.a* treated group (*n* = 5 biological replicates *per* group). Data were presented as the mean ± SEM. Comparisons were performed by unpaired, two-tailed *t* test. **f** Representative images of Lgr5 staining (green) and DAPI staining (blue) in colonoids with 3 independent experiments. The number of Lgr5^+^ cells in each crypt was counted and represented relative to PBS-treated group. *n* = 7 random field of view *per* group in 3 independent experiments. Scale bar, 50 μm. Data were presented as the mean ± SEM. Comparisons were performed by unpaired, two-tailed *t* test. *p < 0.05, **p < 0.01, ***p < 0.001. Source data and exact *p* value are provided as a Source data file.

To further explore the ISC-promoting effect of *B. adolescentis*, we cultured the primary colonoids derived from C57BL/6 mice. Cultured colonoids were treated with heat-inactivated *B. adolescentis* starting on day 3, and the growth parameters of colonoids were evaluated on day 3, 5 and 7, respectively (Fig. 4a). Morphologically, the diameter of colonoids was larger and the proportion of budding colonoids became higher treated with *B. adolescentis* (Fig. 4b, c). These morphologic changes were verified with a significant increased proportion of EdU-positive cells (Fig. 4d). The Lgr5 expression was significantly upregulated with *B. adolescentis* (Fig. 4e, f), which indicates the promoting effect of *B. adolescentis* on the proliferation of ISCs in vitro. Similar promotion effects of ISCs regeneration were also observed in the isolated colonic crypts from G3 *Terc*^-/-^ mice or 12-month-old mice treated with *B. adolescentis* (Supplementary Fig. 4).

We then successfully isolated crypts from G3 *Terc*^*-/-*^ mice model or 12-month-old mice to perform a co-cultured assay with heat-inactivated *B. adolescentis* (Supplementary Fig. 4a). Improvement in budding rate and diameter were found on day 7 in *B. adolescentis* co-cultured system (Supplementary Fig. 4b–e). Furthermore, the expressions of *Lgr5* gene were significantly upregulated by *B. adolescentis* treatment in both models (Supplementary Fig. 4f, g). Collectively, these results confirmed that *B. adolescentis* could promote the regenerative potential of colonoids in vitro.

To testify the specificity of *B. adolescentis* effects, we evaluated the generation of colonoids treated with other well-known commensal bacteria, including *Bifidobacterium pseudolongum* (*B.p, Bifidobacterium* genus), *Lactobacillus johnsonii* (*L.j*, a non-*Bifidobacterium* genus probiotic) and *E. coli* (a common commensal bacterium), as the controls (Supplementary Fig. 5). Our results demonstrated that the observed effects in colonoids were specifically attributable to the presence of *B. adolescentis*, thereby highlighting the distinctive transitional potential of this bacterium.

### Heat-inactivated *B. adolescentis* induced ISCs proliferation was regulated with Wnt/β-catenin signaling

**Fig. 5:**
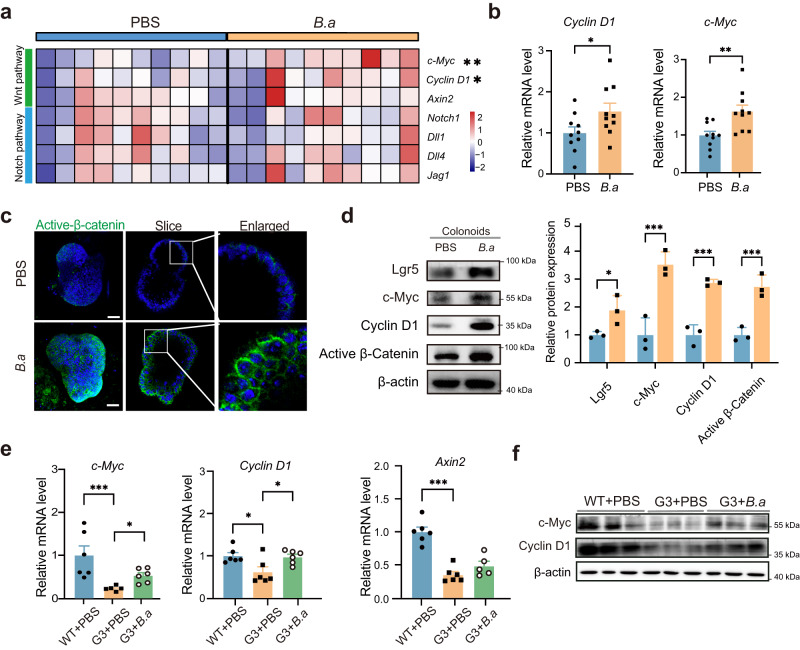
Wnt/β-catenin pathway involved in heated-inactivated *B. adolescentis* induced ISCs proliferation. **a** Heatmap visualization of a series of Wnt/β-catenin and Notch pathway genes in PBS and *B. adolescentis* treated colonoids. Asterisk denotes statistical significance. Source data and exact *p* value are provided as a Source data file. **b** Relative mRNA levels of *Cyclin D1* and *c-Myc* were shown. *n* = 10 individual experiments *per* group. Data were represented as mean ± SEM. Comparisons were performed by unpaired, two-tailed *t* test. **c** Representative images of immunostaining staining with DAPI (blue) and active β-catenin (green) in colonoids. Scale bar, 25 μm. *n* = 3 independent experiments. **d** Immunoblot of Cyclin D1, c-Myc and active β-catenin in colonoids from PBS or *B. adolescentis* treated group. *n* = 3 independent experiments. Data were represented as mean ± SEM. Comparisons were performed by Two-way ANOVA followed by Two-stage linear step-up procedure of Benjamini, Krieger and Yekutieli. **e** The expression of c-*Myc*, *Cyclin D1* and *Axin2* genes were detected by qPCR from WT + PBS, G3 *Terc*^-/-^ + PBS and G3 *Terc*^-/-^ + *B.a* mice group. *n* = 6 animals *per* group. Data were represented as mean ± SEM. Comparisons in *Cyclin D1* and *Axin2* were performed by One-way ANOVA analysis followed by Tukey’s multiple comparisons test and in c-*Myc* were performed by Kruskal–Wallis test followed by Two-stage linear step-up procedure of Benjamini, Krieger and Yekutieli. test. **f** The protein level of c-Myc, Cyclin D were detected by immunoblot from WT + PBS, G3 *Terc*^-/-^ + PBS and G3 *Terc*^-/-^ + *B.a* mice group. *n* = 3 animals *per* group. *p < 0.05, **p < 0.01, ***p < 0.001. Source data and exact *p* value are provided as a Source data file.

Wnt/β-catenin signaling and Notch signaling were reported to be involved in regulating the proliferation and differentiation of ISCs^18,19^. We examined the mRNA levels of genes involved in those two signaling pathways in the colonoids co-cultured with heat-inactivated *B. adolescentis* or PBS control. The results demonstrated that *B. adolescentis* significantly upregulated the levels of *c-Myc* and *Cyclin D1*, which were involved in the Wnt/β-catenin signaling (Fig. 5a, b). Furthermore, higher active β-Catenin fluorescence intensity was observed in the *B. adolescentis* group (Fig. 5c). Protein expression levels of Cyclin D1, c-Myc and active β-Catenin were also significantly upregulated in the *B. adolescentis*-treated group (Fig. 5d). To validate the in vitro findings, the expression of *Cyclin D1* and *c-Myc* were detected in *B. adolescentis*-treated G3 *Terc*^*-/-*^ mice. We also observed that *B. adolescentis* could activate the Wnt/β-catenin pathway (Fig. 5e, f).

**Fig. 6:**
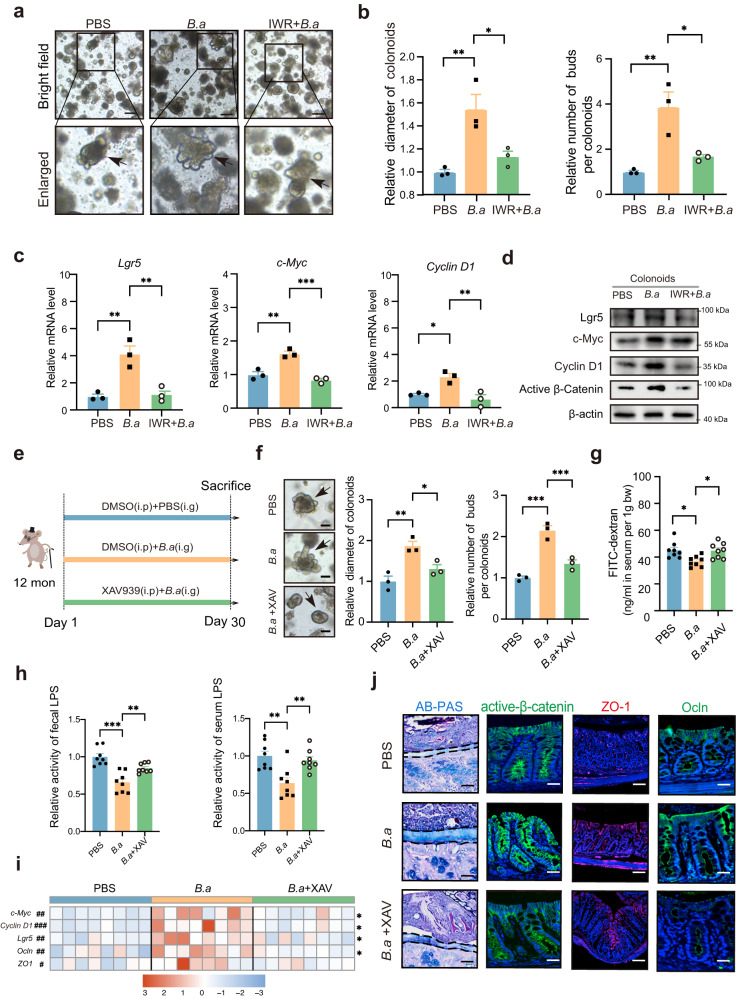
Wnt/β-catenin pathway involved in *B. adolescentis*-induced ISCs proliferation. **a**, **b** Representative images in organoid-forming capacity and size of colonoids quantified from PBS, *B.a* and IWR + *B.a* group. Scale bar, 100 μm. *n* = 3 independent experiments *per* group. Data were represented as mean ± SEM and represented relative to PBS control. Comparisons were performed by One-way ANOVA analysis followed by Tukey’s multiple comparisons test. **c** The expression of Lgr5 and Wnt/β-catenin target genes were detected by qPCR from PBS, *B.a and* IWR + *B.a* treated group. *n* = 3 independent experiments. Data were represented as mean ± SEM. Comparisons were performed by One-way ANOVA analysis followed by Tukey’s multiple comparisons test. **d** The protein level of Lgr5 and Wnt/β-catenin target genes were detected by immunoblot from PBS, *B.a and* IWR + *B.a* treated group. **e** Schematic diagrams of inhibition experiment in vivo from PBS, *B.a*, *B.a* + XAV group, *n* = 8 animals *per* group. **f** Representative images in organoid-forming capacity and size of colonoids quantified from PBS, *B.a*, *B.a* + XAV group on day 7. Arrows indicate crypt domains. Scale bar, 100 μm. Data was from 3 independent experiments with a total of *n* = 8 mice *per* group. Data were represented as mean ± SEM and represented relative to PBS control. Comparisons were performed by One-way ANOVA analysis followed by Tukey’s multiple comparisons test. **g** FITC-dextran concentration in the serum in PBS, *B.a* and *B.a* + XAV group mice. *n* = 8 animals *per* group. Data were represented as mean ± SEM. Comparisons were performed by One-way ANOVA analysis followed by Tukey’s multiple comparisons test. **h** Relative LPS levels in the fecal and serum samples in PBS, *B.a* and *B.a* + XAV mice. *n* = 8 animals *per* group. Data were represented as mean ± SEM. Comparisons were performed by One-way ANOVA analysis followed by Tukey’s multiple comparisons test. **i** Heatmap visualization of a series of (*c-Myc, Cyclin D1, Lgr5, Ocln, ZO-1*) genes in PBS, *B.a* and *B.a* + XAV group. Pound key (#) denotes statistical significance between PBS and *B.a* group, asterisk (*) denotes statistical significance between *B.a* and *B.a* + XAV group. *n* = 8 animals *per* group. Source data are provided as a Source Data file. Comparisons were performed by One-way ANOVA analysis followed by Tukey’s multiple comparisons test. **j** Representative image of AB-PAS staining and immunofluorescence image of active-β-catenin, ZO-1 and Ocln in the colon from indicated mice. *n* = 8 animals *per* group. Scale bar, 50 μm, *n* = 8 animals *per* group. *p < 0.05, **p < 0.01, ***p < 0.001. ^#^*p* < 0.05, ^##^*p* < 0.01, ^###^*p* < 0.001. Source data and exact *p*-value are provided as a Source data file.

To further confirm that the ISC-promoting effect of *B. adolescentis* was related to the activation of the Wnt/β-catenin signaling, the Wnt inhibitor IWR-1-endo (IWR) was added into the colonoids-bacteria co-culture system. We found that the enhanced organoid-forming capacity induced by *B. adolescentis* significantly declined in the presence of IWR (Fig. 6a, b). The mRNA and protein expression of *Lgr5* and Wnt/β-catenin pathway-related genes were inhibited by the addition of IWR (Fig. 6c, d).

To testify whether Wnt/β-catenin signaling is involved in vivo process, XAV939, a pharmacological inhibitor for Wnt/β-catenin signaling, was injected intraperitoneally (i.p.) once per day to the 12-month-old mice treated with heat-inactivated *B. adolescentis* (Fig. 6e). The organoid-formation capacity of colonic crypts and the intestinal barrier function examination after 30 days treatment suggested that XAV939 significantly blocked the enhanced effect induced by *B. adolescentis* (Fig. 6f, g). Increased fecal and serum LPS levels, decreased expression of intestinal-integrity-related genes (ZO-1 and *Ocln*), stemness-related genes *Lgr5* and Wnt target genes (*c-Myc*, *Cyclin D1*) in the XAV939-treated group also confirmed the vital role of Wnt/β-catenin signaling in the process of *B. adolescentis* induced ISCs proliferation (Fig. 6h–j).

### Paneth-like cells were involved in the ISC-promoting effect of heat-inactivated *B. adolescentis*

**Fig. 7:**
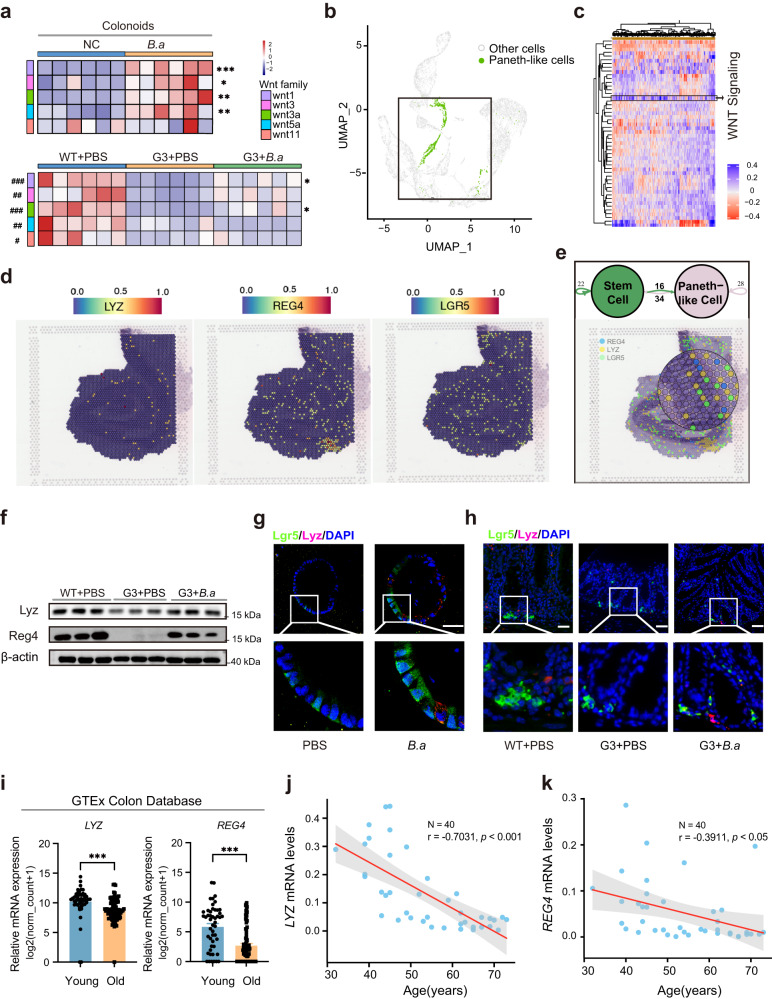
Paneth-like cells were involved in ISC-promoted regulation of *B. adolescentis*. **a** Heatmap visualization of a series of Wnt family genes in PBS or *B. adolescentis* treated colonoids (above). Comparisons were performed by unpaired, two-tailed *t* test. Asterisk denotes statistical significance. *n* = 6 individual experiments *per* group. Heatmap visualization of a series of Wnt family genes in WT + PBS, G3 *Terc*^-/-^ + PBS and G3 *Terc*^-/-^ + *B.a* mice group (below). *n* = 6 individual experiments *per* group. Comparisons were performed by One-way ANOVA analysis followed by Tukey’s multiple comparisons test. Pound key (#) denotes statistical significance between WT + PBS and G3 *Terc*^-/-^ + PBS group, asterisk (*) denotes statistical significance between G3 *Terc*^-/-^ + PBS and G3 *Terc*^-/-^ + *B.a* group. **b** Paneth-like cells (green dots) cluster in human single-cell transcriptome data (GSE125970). **c** Gene set variation analysis (GSVA) was performed in the Paneth-like-cell cluster from the colon tissues. Wnt/β-catenin signaling pathway was highlighted. **d** Integration of LYZ, REG4 and LGR5 gene expression in human colon spatial transcriptomics profiles on Visium datasets (GSE158328). **e** Cell communication between PLCs and ISCs in the single-cell transcriptome (top), and validation of the co-localization of PLCs and ISCs cells in spatial transcriptomics (bottom). **f** Western blot results of Lyz and Reg4 in WT + PBS, G3 *Terc*^-/-^ + PBS and G3 *Terc*^-/-^ + *B.a* mice. *n* = 3 animals *per* group. **g** Immunofluorescence staining of Lgr5 (green) and Lyz (red) in colonoids treated with *B. adolescentis* or PBS as a control. Scale bar, 100 μm. *n* = 3 biological replicates *per* group. **h** Immunofluorescence staining of Lgr5 (green) and Lyz (red) in the colon from WT, G3 *Terc*^-/-^ + PBS and G3 *Terc*^-/-^ + *B.a* mice. Scale bar, 50 μm. *n* = 6 animals *per* group. **i** Relative mRNA levels of LYZ and REG4 gene in normal colon tissues in the young group (20-39 years, *n* = 49 individuals) and old group (60–79 years, *n* = 90 individuals) from GTEx database. Data were represented as mean ± SEM. Comparisons were performed by unpaired, two-tailed *t* test. **j**, **k** Correlation analysis of the expression of LYZ and REG4 with age in human colonic tissues with different ages (*n* = 40, Spearman *r* = −0.7031, *p* < 0.001 and *r* = −0.3911, *p* < 0.05). Comparisons were performed by Pearson’s correlation analysis. *p < 0.05, **p < 0.01, ***p < 0.001. ^#^*p* < 0.05, ^##^*p* < 0.01, ^###^*p* < 0.001. Source data and exact *p* value are provided as a Source data file.

Previous studies have revealed that Paneth cells, which were adjacent to the small intestinal Lgr5^+^ stem cells in crypts and secreted several crucial ISC-supporting factors belonging to the Wnt family, contributed to the proliferation of Lgr5^+^ stem cells and intestinal homeostasis^20^. The existence of colonic Paneth-like cells (PLCs) has recently been reported to support colonic Lgr5^+^ stem cells^21,22^. In colonoids and colon samples, we found that the mRNA levels of *Wnt1* and *Wnt3a* were significantly increased with the *B. adolescentis* intervention (Fig. 7a). Therefore, we tried to explore whether Paneth-like cells were involved in the heat-inactivated *B. adolescentis-*induced ISCs proliferation. We obtained single-cell transcriptome data of human small and large intestines from the Gene Expression Omnibus Database (GEO: GSE125970), defined PLCs with reported markers (*LYZ, CA4, CA7*, and *SPIB*), and identified the PLC cluster in the colon (Fig. 7b and Supplementary Fig. 6a, b). We then applied Gene Set Variation Analysis (GSVA) to independently calculate the signature enrichment scores of individual single cells. Results showed a high level of Wnt/β-catenin signaling in colon tissues (Fig. 7c). We assessed the intracellular communication by “iTALK” algorithms and found 50 paired receptor–ligand interactions between PLCs cells and ISCs cells (Supplementary Fig. 6c). Such frequent interactions were further confirmed in tissue structures from colon spatial transcriptomics (GSE158328) which marked by *LGR5*, *LYZ* and *REG4* (Fig. 7d, e).

We then examined the mRNA levels of reported PLCs-related genes (*Lyz*, *Reg4*, *Car4*,*c-Kit*, *Bip1* and *Cd24)* in colonoids, and found that *B. adolescentis* significantly upregulated the mRNA expression of these genes (Supplementary Fig. 6d). The protein levels of Lyz and Reg4 also significantly increased with heat-inactivated *B. adolescentis* intervention in the colon tissues from G3 *Terc*^*-/*-^ mice (Fig. 7f). Importantly, the localization of Lyz and Lgr5 were confirmed by immunofluorescence staining in colonoids and colon tissues, which demonstrated that *B. adolescentis* might increase the number of PLCs located next to Lgr5^+^ ISCs (Fig. 7g, h).

Lastly, we compared the mRNA levels of PLCs-related genes (*LYZ*, *REG4*, *DAFA6* and *CD24*) between young (20-39 years) and old (60-79 years) group from GTEx colon database^21,23–26^. The mRNA levels of PLCs-related genes significantly declined in the aged populations (Fig. 7i and Supplementary Fig. 6e). We also observed the expression of *LYZ* and *REG4* both negatively correlated with age in colon biopsies (Fig. 7j, k).

### Isolation and identification of potential functional components of heat-inactivated *B. adolescentis*

**Fig. 8:**
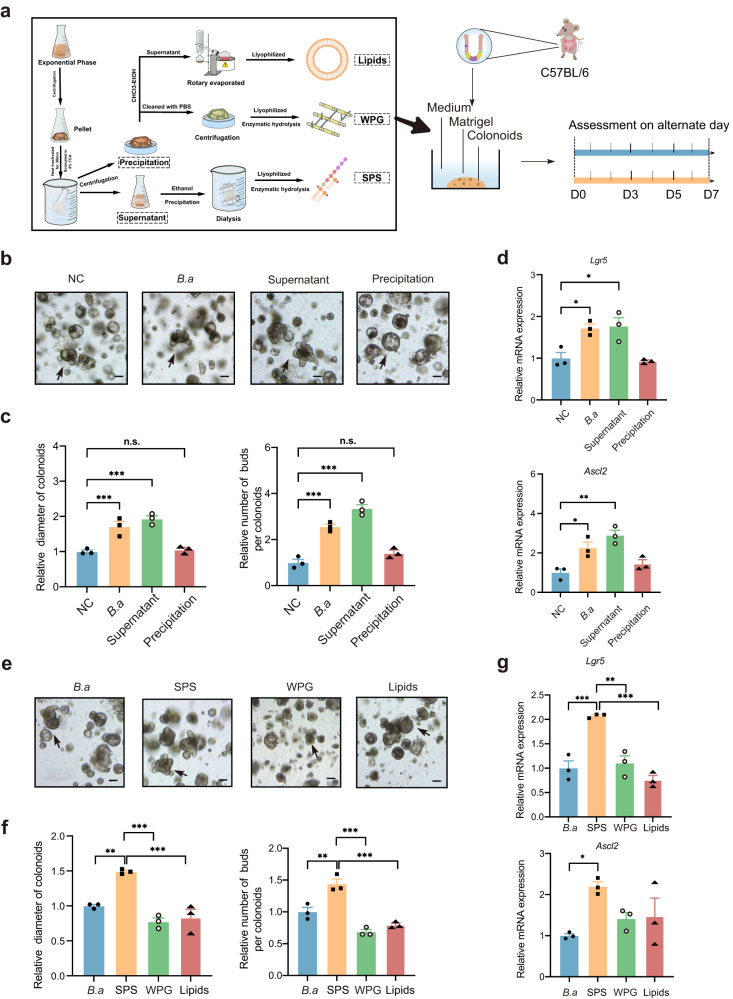
Identification of crude active components in heated-inactivated *B. adolescentis*. **a** Schematic diagrams of crude extraction and intervention about *B. a*. **b**, **c** Representative images in organoid-forming capacity and size of colonoids quantified from NC, *B.a*, supernatant and precipitation group on day 7. Scale bar, 100 μm. Arrows indicate crypt domains. *n* = 3 independent experiments. Data were represented as mean ± SEM and represented relative to NC control. Comparisons were performed by One-way ANOVA analysis followed by Tukey’s multiple comparisons test. **d** The expression of *Lgr5* and *Ascl2* genes were detected by qPCR from NC, *B.a*, supernatant and precipitation group. *n* = 3 independent experiments. Data were represented as mean ± SEM. Comparisons were performed by One-way ANOVA analysis followed by Tukey’s multiple comparisons test. **e**, **f** Representative images in colonoid-forming capacity and size of colonoids quantified from *B.a*, SPS, WPG and Lipids group on day 7. Scale bar, 100 μm. Arrows indicate crypt domains. *n* = 3 independent experiments. Data were represented as mean ± SEM and represented relative to NC control. Comparisons were performed by One-way ANOVA analysis followed by Tukey’s multiple comparisons test. *n* = 3 independent experiments. **g** The expression of *Lgr5* and *Ascl2* genes were detected by qPCR from *B.a*, SPS, WPG and Lipids group. *n* = 3 independent experiments Data were represented as mean ± SEM. Comparisons were performed by One-way ANOVA analysis followed by Tukey’s multiple comparisons test. *p < 0.05, **p < 0.01, ***p < 0.001, n.s. not significant. Source data and exact *p* value are provided as a Source data file. SPS soluble polysaccharides, WPG whole peptidoglycan.

In order to gain insight into the effective components of heat-inactivated *B. adolescentis*, we extracted and separated crude components of *B. adolescentis* as indicated in the schematic diagram (Fig. 8a). The organoid size was significantly increased when co-cultured with isolated supernatant of heat-inactivated *B. adolescentis*, while cleavage products of precipitation did not have this effect (Fig. 8b–d). Based on these findings and the latest reported bacteria functional components^27–29^, we subsequently cultured the colonoids with crude compositions from *B. adolescentis*, including soluble polysaccharides (SPS), whole peptidoglycan (WPG, insoluble polysaccharides) and lipids. Results showed that only SPS could promote the proliferation of colonoids and increase the expression of the stemness-related gene (*Lgr5* and *Ascl2*), which indicated that SPS might act as functional ISC-promoting components of *B. adolescentis* rather than lipids and peptidoglycans (Fig. 8e–g).

We subsequently tried to characterize the fundamental structure and composition of the polysaccharide (as depicted in the flowchart, Supplementary Fig. 7a). SEM (Scanning electron microscope) revealed an uneven, irregular, tile-like surface morphology of the SPS, with a molecular weight of approximately 20 kDa ascertained via GPC as illustrated (Supplementary Fig. 7b, c). FT-IR spectra of SPS exhibited a variety of typical absorption peaks of polysaccharides. These results suggested that SPS had the typical chemical bond structures of polysaccharides (O-H, C-H, C = O and C-OH vibrations) (Supplementary Fig. 7d). Finally, a monosaccharide composition analysis revealed that SPS were mainly consisted of mannose (Man), glucose (Glc), galactose(Gal), and glucuronic acid (Glc-UA) (Supplementary Fig. 7e–g).

### Heat-inactivated *B. adolescentis* demonstrated a comparable effect as live *B. adolescentis* in promoting ISCs

To further investigate whether the inactivated or live state of *B. adolescentis* exerted its beneficial effects on the senescent colon, we compared the regeneration ability of the colonoids after treatment with heated-inactivated or live *B. adolescentis*. In conjunction with the bacterial plating, the results showed that live *B. adolescentis* demonstrated a similar ability to promote intestinal stem cells as heat-inactivated *B. adolescentis* in vitro (Supplementary Fig. 8a–c). Subsequently, we proceeded with in vivo experimental validation and confirmed that a 30-day intervention with live or heat-inactivated *B. adolescentis* in 12-month-old mice both improved the intestinal barrier integrity corresponding to the increased level of ISCs (Supplementary Fig. 8d–i).

To confirm that *B. adolescentis*-derived SPS was not artificial production during heat inactivation, we characterized the fundamental thermal stability of SPS (Supplementary Fig 9a–c). The subsequent characterization experiments of SPS derived from heat-inactivated and live *B. adolescentis* showed a high degree of similarity (Supplementary Fig. 9d–i). Finally, the SPS derived from heat-inactivated and live *B. adolescentis* also indicated a comparable efficacy in promoting colonoid-forming (Supplementary Fig. 9j–l). The consistent physicochemical properties and biological activity strongly suggest that SPS is the natural component of *B. adolescentis*.

## Discussion

**Fig. 9:**
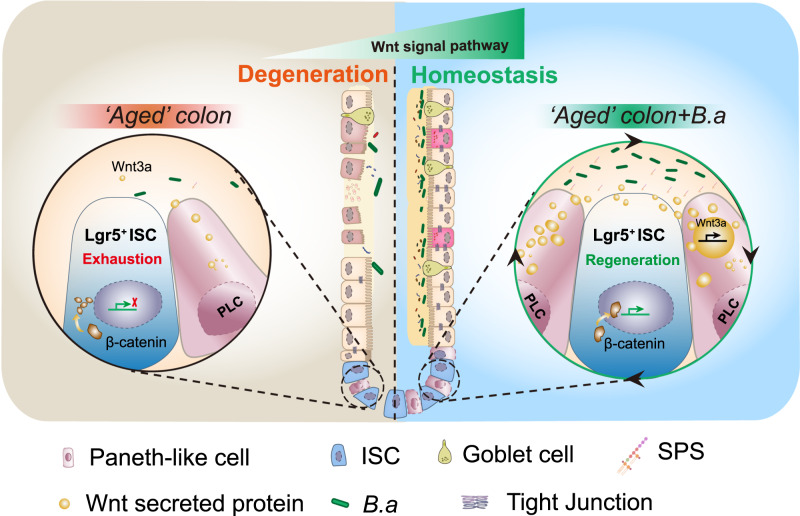
A model of the proposed mechanism by which *B. adolescentis* ameliorates colon senescence. The proposed scheme shows that *B. adolescentis* ameliorates senescent colon and intestinal integrity by promoting ISCs regeneration via the PLCs-mediated Wnt/β-catenin pathway. SPS soluble polysaccharides, ISC intestinal stem cells, *B.a* *Bifidobacterium adolescentis*.

As a vital center for signal transmission and metabolic homeostasis maintenance, the healthy intestine is an important determinant of lifespan^30^. Gut-associated *Bifidobacterium* has been shown to exert beneficial effects on healthy aging. Our recent studies revealed that heat-inactivated *B. adolescentis* could remit intestinal inflammation and improve healthy aging^12,31^. However, there is limited knowledge regarding the impact of *B. adolescentis* on ISCs. In this study, we demonstrated that heat-inactivated *B. adolescentis* could ameliorate colon senescence by promoting the regeneration of ISCs in vivo and in vitro through the PLCs-mediated Wnt/β-catenin pathway (shown in the schematic diagram, Fig. 9).

In this study, we demonstrated that the impaired intestinal barrier function and declined ISCs are concomitant with the decreased abundance of *B. adolescentis* during aging. Previous studies have characterized senescent colon with thinner mucosal layers, distorted villi, deregulated goblet cells, impaired tight junctions between adjacent enterocytes and exhausted stem cells^2,32–36^. Several studies have linked the alteration in bacterial populations with aging processing. *Bifidobacterium longum* and *Bifidobacterium breve* were generally dominant in infants, whereas *Bifidobacterium catenulatum* and *Bifidobacterium adolescentis* were more prevalent in the adult than the old^37^. Meanwhile, the aging process also increases the proportion of specific Gram-negative bacteria, such as *Enterobacteriaceae*, which produce pro-inflammatory lipopolysaccharides (LPS)^38,39^. Gut microbiota-derived LPS, could be detected in serum when intestinal integrity is compromised during aging. LPSs from different bacteria have distinct effects on gut-barrier function, which indicates bacterial species specificity^40,41^. Therefore, the detection of pathogenic LPS is of substantial importance. Our study revealed that heat-inactivated *B. adolescentis* could reduce the elevated levels of LPS during the aging process.

We also validated the protective effect of heat-inactivated *B. adolescentis* on Lgr5^+^ ISCs. Self-renewal and differentiation of ISCs are essential for intestinal epithelial integrity, which were related to the aging process^32,42–44^. Recent studies have adopted ISCs-targeted strategies to rescue senescent or damaged intestines from gene regulation to microbial active ingredients^34,36,45–47^. We established the colonoids and *B. adolescentis* co-cultured model and found that *B. adolescentis* significantly enhanced the organoid-forming capacity of the colonic crypts derived from aged mice. The Wnt/β-catenin signaling pathway serves a vital role to promote the regeneration of ISCs^48^. Several studies have proved that microbial components and other probiotics could activate the Wnt/β-catenin signaling. Supplementary lactate could promote the proliferation of Lgr5^+^ ISCs and epithelial development by stimulating the Wnt/β-catenin signaling in Paneth and intestinal stromal cells^45^. *Lactobacillus reuteri* could increase Wnt3, Lrp5 and β-catenin expression to support the proliferation of intestinal epithelia^17,49^. The reduced regenerative capacity of ISCs upon aging resulted from a declining niche (Paneth or mesenchyme) secretion of Wnt signals, and reactivating canonical Wnt signaling ameliorated aging-associated phenotypes of ISCs from organoid to human ISCs^19^. Our study demonstrated that the promotion effect of *B. adolescentis* on the senescent colon is related to the regeneration of ISCs facilitated by the activation of Wnt/β-catenin signaling.

Recently, the PLCs were reported as essential supporting cells for the colonic stem cell niche and might represent the Paneth cell equivalents^20,21,25^. PLCs were confirmed in the rat ascending colon and human fetal large intestine^22,24^. We analyzed spatial transcriptomics data and single-cell RNA sequencing data from human intestine bio-samples and demonstrated that the PLCs in the colon had significant interaction with ISCs. PLCs highly expressed niche-factor-encoding genes including EGF, Wnt3, Notch, and PDGF ligands, which activate the ISCs proliferation through Wnt/β-catenin signaling pathway^22^. Our results indicated that *B. adolescentis* administration might stimulate the expression of maker genes of Paneth-like cells and Wnt family-secreted proteins, such as Wnt3 and Wnt3a, which could promote ISCs.

It is interesting to explore how heat-inactivated *B. adolescentis* activate the PLCs. We found that it was the isolated supernatant but not precipitation products that played the rejuvenated effect on ISCs. Moreover, our further exploration of the functional components of heat-inactivated *B. adolescentis* showed that only soluble polysaccharides (SPS) exerted the ISC-promoting effect. More recently, it has been appreciated that some bacterial-derived bioactive polysaccharides have health benefit effects^50–52^. Several studies have mentioned that the cell surface β-glucan or galactan (CSGG) polysaccharide of *Bifidobacterium bifidum* (*B. bifidum*), another species belonging to genus *Bifidobacterium*, shows immunomodulatory potential and displays great suppressive capacity toward colitis^51^. These results showed SPS are the potential active ingredients to promote ISCs proliferation.

Considering the similarity with Paneth cells, we assumed that PLCs might express similar pattern recognition receptors (PRR), such as Toll-like receptors (TLRs) and NOD-like receptors. However, classical receptors were not included in the marker genes of PLC-related expression profiling^22^, which suggested that PLCs might exhibit different mechanisms for responding to the external environment from Paneth cells. The detailed regulatory mechanism of PLCs would be a promising project in the future.

It has been traditionally believed that probiotics must be alive to confer health benefits to the host. Certain components on the cell surface of postbiotics, such as polysaccharides, peptidoglycans, surface proteins, and phosphoric acid, play a role in their interaction with the host and contribute to the health benefits^53–55^. Previous study also suggested polysaccharides exert physical and chemical properties that can influence the immune system^56^. In our study, one specific postbiotic derived from *B. adolescentis*, which remains stable even when the *B. adolescentis* is heated, seems to play a vital role in ameliorating colon senescence. It is noteworthy that the utilization of heat-inactivated *B. adolescentis* may impose limitations on our exploration of the other bioactivity aspects related to enzymes and metabolites. Meanwhile, the potential advantages of live probiotics remain worthy of exploration, including the direct interaction of live probiotics with the host immune system and gut microbiota.

In conclusion, our study presented that heat-inactivated *B. adolescentis* could remit the the colon senescence by promoting ISCs regeneration via Wnt/β-catenin pathway. We also simultaneously identified SPS as a potentially effective component SPS of heat-inactivated *B. adolescentis*. Our findings provide insights into the development of postbiotics for the treatment of age-related intestinal diseases.

## Methods

All research conducted for this study complies with the relevant ethical regulations. Study protocols were approved by the Animal Ethical Committee of Zhejiang University for all animal experiments and the human patient study was approved by the Ethics Committee of Sir Run Run Shaw Hospital, School of Medicine, Zhejiang University.

### GTEx colon tissues and expression data

The Genotype-Tissue Expression (GTEx) was a project that collected multiple different human tissues from hundreds of donors and performed genotyping, gene expression profiling, whole-genome sequencing, and RNA sequencing (RNA-seq) analyses. The expression data (v6, October 2015 release) for colon specimens were obtained from the GTEx portal website (http://www.gtexportal.org). The data was generated using RNA-seq with tissues initially sampled from colon regions. From the downloaded data, we extracted the expression values for protein-coding genes. The donor’s information on gender, body mass index (BMI), and age were also downloaded.

### GMrepo database analysis

Microbial relative abundance information in health among different age groups was obtained from the GMrepo database^57^. GMrepo is a curated and annotated human gut metagenomic data repository about microbiota. Specific bacteria relative abundance was calculated at the species level for each sample.

Using GMrepo RESTful APIs for R (version 3.6.3, https://www.r-project.org) and RStudio (version 1.3.1093, https://www.rstudio.com) software, we obtained relative abundances of *B. adolescentis* in adult healthy people, then we evaluated the quality by BMI between 18.5 and 23.9, we also filter some samples by acquiring abstraction from related publications such as fecal microbiota transplantation. The linear relationship was calculated by Spearman’s correlation coefficient test between *B. adolescentis* relative abundance and age, *B. adolescentis* relative abundance was also extracted and plotted at the same time with R basic packages.

### CuratedMetagenomicData database analysis

The CuratedMetagenomicData is a public and timely updated R package in the Bioconductor Experiment-Hub platform^58^. The provided extensive metadata information was processed by a uniform bioinformatic analysis pipeline. The CuratedMetagenomicData repository includes 30 kinds of disease phenotypes from more than 25 different studies and taxonomic profiles of more than 6000 human microbiome samples from different body sites. More importantly, the database contains experimental details about the region (or country), disease status, age, gender, BMI and antibiotic usage history.

Since we mainly focus on specific species in different age groups, we selected a subset of CuratedMetagenomicData, which is filtered by body site in stool, with antibiotic usage at none. Subsequently, we removed samples with an age less than 20. Thereby this dataset is filtered with metadata information using R. Thereafter, we first evaluate the top species abundance distribution in different age groups, then a linear relationship determined by Spearman’s correlation coefficient test between *B. adolescentis* relative abundance and age, *B. adolescentis* relative abundance was also extracted and plotted at the same time with R basic packages.

### *B. adolescentis* treatment and mice experiments

All animal studies were performed in accordance with the guidelines of the Institutional Animal Use and the Animal Experimentation Ethics Committee at Zhejiang University. Heterozygous telomerase RNA component *Terc* knockout mice (G0 *Terc*^+/-^) in the C57BL/6 background were gifted by Dr. Song^12^, Generation one knockout animals (G1 *Terc*^-/-^) were derived from G0 *Terc*^+/-^ mice and finally generate G3 *Terc*^-/-^ for experiments. 6–8-week-old wild-type (WT + PBS, *n* = 11, 8 males and 3 females) littermates were administrated intragastrically with PBS as the control group. 6–8-week-old *Terc*^-/-^ G3 littermates were randomly assigned to the PBS group (G3 + PBS, *n* = 9, 5 males and 4 females) and *B. adolescentis* group (G3 + *B.a*, *n* = 12, 7 males and 5 females) administrated intragastrically with heat-inactivated *B. adolescentis* until natural death or sacrifice at 7 months old. For other animal experiments, C57BL/6 mice (3-month-old and 12-month-old, male) were used in the experiment. the number of each experiment is listed in the figure legends. All mice were maintained at a specific pathogen-free (SPF) level animal facility in Sir Run Run Shaw Hospital with a room temperature of 20–22 °C and humidity (45–65 °C). Mice were provided with a standard laboratory diet and water freely in ventilated cages with a 12-h light/12-h dark circadian cycle.

### Preparation of *B. adolescentis* and control bacterial species

*B. adolescentis* ATCC15703 was purchased from American Type Culture Collection (ATCC, USA, *Bifidobacterium adolescentis* Reuter ATCC 15703), and cultured in anaerobic modified reinforced clostridium medium (RCM, ATCC medium 2107) under an atmosphere of 10% H_2_, 10% CO_2_, and 80% N_2_ (AW500SG anaerobic workstations; ELECTROTEK, England) for 48 hours. The cultures were centrifuged at 2500 × *g* for 5 min at 4 °C, washed twice with sterile anaerobic PBS, and resuspended for a final concentration of 1×10^9^ CFU/200 µl under strictly anaerobic conditions. *B. adolescentis* was heat-inactivated in 95°C water bath for 15 min before experiment treatment. Each mouse was administrated intragastrically with 200 μl sterile PBS or heat-inactivated *B. adolescentis* every other day. *Lactobacillus johnsonii* (*L.j*) and *Bifidobacterium pseudolongum* (*B.p*) was isolated and gifted by Zhejiang Academy of Agricultural Sciences and confirmed on the species level by 16S ribosomal RNA sequencing (V4 sequences). *E. coli* (*E.c*) strain MG1655 (Biobw, China) was used as a negative control. *E. coli* was cultured in Luria-Bertani (LB) Medium (A507002 Sangon Biotech, China) at 37 °C. *L.j and B.p* were cultured in De Man, Rogosa and Sharpe (MRS) Medium (HB0384-5, hopebio, China) under an atmosphere of 10% H_2_, 10% CO_2_ and 80% N_2_ (AW500SG anaerobic workstations; ELECTROTEK, England).

### Isolation process of *B. adolescentis* crude extraction

The extraction process of crude product was based on previous literature reports^27,28,51,59,60^. Briefly, *B. adolescentis* were cultured to the exponential growth phase. After centrifugation, the bacterial pellet was washed repeatedly. Permeability of the *B. adolescentis* was increased by inactivation and sonication in 4% TCA. In preliminary experiments, cleavage products were centrifuged to generate precipitation and supernatant. Subsequently, Further crude extraction was carried out based on preliminary experiments. Soluble polysaccharides (SPS) were extracted by the water extraction and alcohol precipitation method. Lipids were extracted by chloroform and methanol (1:1) by stirring for 24 h at room temperature. Whole peptidoglycan (WPG) was centrifuged, washed, and digested by proteases and nucleases. All the crudes were enriched by rotary evaporation or lyophilization in the end. As for nucleic acids and proteins, we performed proteases (10401ES60, Yeasen, China) or nucleases (20156ES25, Yeasen, China) treated experiments of the *B. adolescentis* lysate.

### H&E staining and immunofluorescence

Colon tissues were fixed in 4% paraformaldehyde. Hematoxylin and eosin (H&E) staining and Periodic acid Schiff (PAS) staining were conducted to assess morphological changes. The paraffin-embedded colon tissues were deparaffinized, rehydrated, retrieved, and antigen retrieval. Tissues were sealed with 3% bovine serum albumin and incubated with primary antibodies: anti-Muc2 antibody (Servicebio, GB14110, 1:500), anti-ZO1 antibody (Servicebio, GB111402, 1:500), anti-Occludin antibody (Servicebio, GB111401, 1:500), anti-Lgr5 (Bioss, bs-20747R,1:100). The slices were then incubated with secondary antibodies (Fdbio, FD0129, 1:500; Fdbio, FD0136,1:500). The images were scanned with Pannoramic Scanner (Pannoramic DESK, 3DHISTECH) and observed with Caseviewer C.V 2.3.

### Fluorescence in situ hybridization (FISH)

Detection of *B. adolescentis* was performed by FISH on the formalin-fixed paraffin-embedded (FFPE) tissues. The probe was synthesized by Guangzhou EXON Biological Technology Co. China and incubated with the section of colon tissue.

### Human sample collection

Colonic tissue samples were obtained from 40 normal people for health examination from Sir Run Run Shaw Hospital of Zhejiang University School of Medicine. Healthy people had no history of diarrhea or use of antibiotics or probiotics in the past two weeks. Fresh tissue samples were collected were immediately frozen and stored at −80°C. All participants provided written informed consent before collection and the Clinical Research Ethics Committee of the Sir Run Run Shaw Hospital of Zhejiang University School of Medicine approved the study protocol (20211103-35). Information on human samples is provided in the Supplementary Table S2.

### Bacterial DNA extraction and quantification

Bacterial genomic DNA from healthy human tissues was extracted using TIANGEN DNA kits (DP304-02, TIANGEN, Beijing). Quantitative real-time PCR was performed in ROCHE LightCycler®480 System (Rotor gene 6000 Software, Sydney, Australia). Each reaction was performed in triplicate with qPCR SYBR Green Master Mix (11198ES08, YEASON, China), The relative abundance levels were calculated using the comparative cycle method (2^−ΔΔCt^). Universal Eubacteria 16s was used as a reference gene. The gene primers were listed in Supplementary Table S1.

### Colonoids culture and treatment

Fresh colon tissues isolated from C57BL/6 mice (3 months old) were opened longitudinally and washed three times in cold phosphate-buffered saline (PBS) containing penicillin–streptomycin to remove content and mucus. Subsequently, the tissues were minced and followed by 40 min incubation in 10 mM EDTA on a rotor at 4 °C. Crypts were sequentially shed by mechanical shearing in PBS with 0.01% BSA by pipette tips. After filtration through a 100 μm of cell strainer, 200 crypts were mixed with 50 μl Matrigel (Corning # 356231) and added as a dome in the center of the warm 24-well plate. Then it was incubated at 37 °C, 5% CO_2_ for 10 min to solidify. Complete Intesticult medium (Stemcell Technologies #06005) containing Y-27632 (10 μM, Selleck,1049) and CHIR (5 μM, Selleck,1253) was added for colonoids growth and replaced every other day. Colonoids were sub-cultured every 5–7 days. After 3–4 days of culture, the best‐looking wells were selected for *B. adolescentis* intervention. We settle on the same number of crypts per group for comparison study.

For in vivo experiments, primary colonoids were cultured from 3-month-old and 12-month-old mice which were administrated intragastrically with *B. adolescentis* or PBS in vivo for one month. Colonoids were photographed daily by an inverted microscope (ZEISS LSM 800, Munich, Germany) for 7 days to evaluate the regenerative growth situation, including the number of de novo crypt buddings per colonoids and the diameter of each colonoids. Image analysis of colonoids growth situation has been processed and analyzed by open-source Fiji(NIH, version:1.2.0), QuPath (Edinburgh, version:0.3.0) and Zen image program(ZEISS LSM 800, Munich, Germany) according to the images. Colonoids from six random non-overlapping images in a well were measured.

For in vitro experiments, primary colonoids of the aged model were derived from G3 *Terc*^-/-^and 12-month-old in vitro and then treated with *B. adolescentis*. For signaling pathway inhibition experiments, co-cultured colonoids systems with *B. adolescentis* were treated with or without IWR-1-endo (10 μM) (IWR, Selleck, S7086) or PBS. Colonoids were continuously and stable cultured until the third day, at which point, they were treated with *B. adolescentis* at MOI of 100. Images were taken on Days 3, 5, and 7. Further measurements for colonoids were performed on Day 7 as described above.

### Colonoid EdU staining

Cell-Light EdU DNA cell proliferation kit (C103101, RiboBio) was used to detect the proliferation of colonoids according to the manufacturer’s instructions. Briefly, colonoids were exposed to 50 μM of EdU for 2 h at 37 °C, then the colonoids were fixed in 4% paraformaldehyde (PFA) for 30 min at room temperature. After permeabilization with 0.5% Triton X-100, the colonoids were reacted with 1× Apollo reaction cocktail (RiboBio) for 30 min. Subsequently, the DNA contents of the colonoids were stained with DAPI for 30 min and visualized under a Laser Scanning confocal microscope (ZEISS LSM 800, Munich, Germany). The numbers of EdU^+^ cells per colonoids were processed and analyzed by open-source Fiji (NIH, version 2.1.0) and QuPath (Edinburgh, version:0.3.0).

### Colonoids immunofluorescence assay

To determine immunocytofluorescence of protein expression, colonoids were fixed with 4% PFA for 15 min at room temperature, permeabilized in PBS containing 0.1% Triton X-100 for 30 min at room temperature, and subsequently blocked in PBS containing 5% BSA at room temperature for 1 h. Colonoids were then incubated with primary antibody overnight at 4 °C in blocking buffer (anti-LGR5, OriGene, TA503316,1:100; anti-active β-Catenin, CST, #8814,1:100; Anti-Lysozyme, Abcam, ab108508,1:100). The colonoids were washed in PBS and incubated with Alexa Fluor 488/594-labeled secondary antibody appropriate (Fdbio, FD0150,1:500; Fdbio, FD0129,1:500) for the respective species for 2 h at room temperature. After removing the antibody, colonoids were counterstained with DAPI (1:1000, Beyotime, C1002) for 30 min at room temperature and viewed under a confocal laser scanning microscope (ZEISS LSM 800, Munich, Germany).

### Western blot analysis

Tissue was weighed and homogenized in RIPA extraction buffer (Solarbio, China). The homogenate was centrifuged at 4 °C for 15 min at 15,000 × *g*, then the supernatant was collected. The protein concentration was quantified with BCA protein assay kits (Meilun, China) according to the manufacturer’s instructions. Proteins were separated by 10% SDS polyacrylamide gel and then transferred onto PVDF membranes. The membranes were blocked with 5% skimmed milk for 1 h and then immunoblotted with primary antibodies: p53 (ab167161, Abcam,1:1000), p21 (sc-6246,Santa Cruz,1:500), Lgr5 (Bioss, bs-20747R, 1:1000), c-Myc (CST, #5605S, 1:1000), Cyclin D1 (CST, # 2978, 1:1000), Active β-Catenin (CST. # 8814, 1:1000), Reg4 (Abclonal, A13129, 1:1000), β-actin (ET1701-80, HUABIO, 1:1000), Gapdh (ET1601-4, HUABIO,1:1000) at 4 °C overnight. Membranes were then incubated with second antibodies (HA1019, HA1006, HUABIO,1:10,000) labeled with HRP at room temperature for 2 h, and bands were visualized using an ECL kit (Fdbio science, China). β-Actin or Gapdh was used as a reference protein.

### RNA extraction and quantitative real-time PCR

RNA was extracted from the tissues or colonoids by TRIzol reagent (Takara, Japan) and reverse transcribed by PrimeScript™ RT reagent Kit (Takara, Japan). qRT-PCR was performed using SYBR Premix Ex Taq (Takara, Japan) in the Light Cycler®480 Real-Time PCR System (Roche) using cDNA. The relative mRNA levels were calculated using the comparative cycle method (2^−ΔΔCt^). β-actin was used as a reference gene. The gene primers were listed in Supplementary Table S1.

### Sequencing by 16S rRNA

Total genome DNA from mice stool samples was extracted with TIANamp Stool DNA Kit (Tiangen Biotech) according to the manufacturer’s instructions. Agarose gels was for Quality controls of DNA. Sequencing libraries were generated usingTruSeq® DNA PCR-Free Sample Preparation Kit (Illumina, USA) following the manufacturer’s recommendations and index codes were added. The library quality was assessed on the Qubit@ 2.0 Fluorometer (Thermo Scientific) and Agilent Bioanalyzer 2100 system. At last, the library was sequenced on an Illumina NovaSeq platform and 250 bp paired-end reads were generated. Bacterial taxonomy determination and data analysis were performed on the online platform of Majorbio Cloud Platform (www.majorbio.com).

### Pharmacological inhibition XAV-939 in animal experiment

12-month-old mice were divided into three groups. Two groups were injected with 250 μl 2.4 mg/ml Wnt inhibitor XAV-939 (MedChemExpress, HY-15147) once per day. One of the injection groups was intragastric administrated intragastrically with heat-inactivated *B. adolescentis* once per day. After a month of treatment, we assessed the levels of activation of the β-catenin signaling pathway and intestinal integrity by Alcian Blue-Periodic Acid Schiff (AB-PAS) staining and immunofluorescence staining. The activation of the β-catenin signaling pathway was also evaluated by qRT-PCR.

### FITC-dextran assay for intestinal integrity

Intestinal integrity was determined by FITC-dextran assay^61,62^. Briefly, 6 μl/g body weight of FITC-conjugated dextran (100 mg/ml in PBS; 4.4 kDa FITC-dextran: FD4, Sigma-Aldrich) was administered to each mouse by intragastric administration. After 4 h, blood was collected from the retro-orbital plexus under pentobarbital anesthesia and centrifuged (10,000 × *g* at 4 °C) for 10 min. Diluted serum was added to a 96-well microplate in duplicate. The concentration of FITC in serum was determined by Spectro photo-fluorometry (Wallac Victor; Perkin-Elmer Life Sciences) with an excitation of 485 nm (20 nm bandwidth) and an emission wavelength of 528 nm (20 nm bandwidth) using serially diluted samples of the FITC-dextran marker as standard.

### Fecal lipopolysaccharide (LPS) load quantification

Levels of fecal lipopolysaccharide (LPS) were quantified by HEK-Blue™ hTLR4 cells^63,64^. HEK-Blue™ hTLR4 cells were kindly provided by Professor Yiqi Wang (Zhejiang Chinese Medical University, China). The cells were cultured in Dulbecco’s Modified Eagle Medium (DMEM) containing 10% heat-inactivated fetal bovine serum at 37 °C and 5% CO_2_. The fecal and serum samples were resuspended and homogenized in PBS, and then the samples were centrifuged at 8000 × *g* for 5 min. The supernatant was serially diluted and applied to HEK-Blue™ hTLR4 cells to find the optimal working concentrations. Gradient dilutions of LPS (Sigma, USA) were used as positive controls for HEK-Blue-mTLR4 cells. After 24 h of stimulation, the cell culture supernatant was applied to QUANTI-Blue™ medium (Invivogen, USA), and the alkaline phosphatase activity was measured at 620 nm after 30 min or 90 min.

### Monosaccharide composition analysis

Approximately 5 mg of the sample was hydrolyzed with trifluoroacetic acid (2 M) at 121 °C for 2 h in a sealed tube. Dry the sample with nitrogen. Add methanol to wash, then blow dry, repeat methanol wash 2–3 times. The residue was re-dissolved in deionized water and filtered through 0.22 μm microporous filtering film for measurement. High-performance anion-exchange chromatography (HPAEC) on a CarboPac PA-20 anion-exchange column (3 by 150 mm; Dionex) with a pulsed amperometric detector (PAD; Dionex ICS 5000+ system) was used to analyzed sample. Thirteen standard monosaccharides, namely fucose (Fuc), rhamnose (Rha), arabinose (Ara), galactose (Gal), mannose (Man), glucose (Glc), xylose (Xyl), mannuronic acid (Man-UA), fructose (Fru), ribose (Rib), guluronic acid (Gul-UA), glucuronic acid (Glc-UA), and galacturonic acid (Gal-UA) were used as the references. Data were acquired on the ICS5000+ (Thermo Scientific), and processed using chromeleon 7.2 CDS (Thermo Scientific).

### Fourier-transformed infrared (FT-IR) spectroscopic analysis

The groups of SPS were performed by FT-IR spectra analysis. The sample of polysaccharides (10 mg) was mixed with KBr (200 mg), pressed into 1 mm thickness and measured by Perkin-Elmer spectrum GX FT-IR system (PerkinElmer, USA) at the range of 4000–400 cm^−1^ wavelength.

### Determination of molecular weight (Mw)

Gel-permeation chromatography (GPC) was used to analyze the Mw of SPS. The sample solution passed through 0.22 μm aqueous membrane and 20 μl of sample solution was injected at a flow rate of 0.6 ml/min (0.02% (w:v) NaN3 as mobile phase), The standard curve based on the retention time of different Mw dextrans and the logarithm of relative molecular mass.

### Scanning electron microscope (SEM) analysis

Samples were fixed and subjected to gold plating. The surface morphology of the SPS sample was observed by a scanning electron microscope (Nova Nano 450. Thermo FEI).

### Statistics and reproducibility

Each experiment was performed with at least three biological replicates. Representative images for fluorescence staining, PAS staining, and H&E staining are shown, each of these experiments included at least three independent samples and was repeated at least three times. The data are presented as the mean ± SEM (Standard Error of Mean) with GraphPad Prism (GraphPad Software 9.0.0, San Diego, CA, USA). Differences between the two groups were analyzed by unpaired, two-tailed Student’s *t* test or two-tailed nonparametric test. Differences between the three groups were analyzed by one-way analysis of variance (ANOVA) with multiple comparisons test. Correlations were performed using the Pearson or Spearman correlation test, and *p* values < 0.05 were considered to be statistically significant. No statistical method was used to predetermine the sample size. The sample sizes (“*n*”) and the statistical tests are described in each figure legend, exact *p* values are provided in the Source Data (*p < 0.05, **p < 0.01, and ***p < 0.001).

### Reporting summary

Further information on research design is available in the Nature Portfolio Reporting Summary linked to this article.

### Supplementary information

Supplementary Information

Reporting Summary

### Source data

Source Data

## Data Availability

The raw 16S rRNA gene sequences data generated in this study have been deposited in the Genome Sequence Archive (GSA) database^65^, under accession code CRA008671^66^. The Spatial transcriptomics and single-cell transcriptome data used in this study are available in the Gene Expression Omnibus (GEO) database under accession code GSE125970. The transcriptome of mice colonic tissue data used in this study are available in the Gene Expression Omnibus (GEO) database under accession code GSE94515. GMrepo database is accessible by link (https://gmrepo.humangut.info/), CuratedMetagenomicData database is accessible by link (https://waldronlab.io/curatedMetagenomicData/), GTEx database can be obtained from the GTEx portal website (http://www.gtexportal.org). Other data from the findings of this study are available from the corresponding author upon request. Source data are provided with this paper.

## Code availability

16S rRNA gene sequence data and single-cell transcriptome data analyses were performed using standard QIIME2 (2017.6) (https://qiime2.org/) and Seurat (4.3.0) (https://satijalab.org/seurat/) analysis pipelines. Analysis R scripts employing R packages are available from https://github.com/Yadong-Qi/Paneth-like-cells-AND-stem-cell-activation.
