# Supplementary material for: Heat-inactivated Bifidobacterium adolescentis ameliorates colon senescence through Paneth-like-cell-mediated stem cell activation
Source: Nat Commun. 2023 Sep 30;14:6121. doi: 10.1038/s41467-023-41827-0 (PMC10542354; doi:10.1038/s41467-023-41827-0)
Supplement: Supplementary file 1 — Supplementary Information [file 41467_2023_41827_MOESM1_ESM.pdf]

Supplementary Information

Heat-inactivated *Bifidobacterium adolescentis* ameliorates colon senescence through Paneth-like-cell-mediated stem cell activation

Yadong Qi[1,2,#], Jiamin He[1,2,#], Yawen Zhang[1,2,3,#], Qiwei Ge[2,4,#], Qiwen Wang[1,2,#], Luyi Chen[3,5], Jilei Xu[1,2], Lan Wang[1,2], Xueqin Chen[1,2], Dingjiacheng Jia[2,4], Yifeng Lin[2,4], Chaochao Xu[2,4], Ying Zhang[2,4], Tongyao Hou[1,2], Jianmin Si[1,2,3,*], Shujie Chen[1,2,3,*], Liangjing Wang[2,3,4,*]

[1] Department of Gastroenterology, Sir Run Run Shaw Hospital, Zhejiang University School of Medicine, Hangzhou, Zhejiang, China.

[2] Institute of Gastroenterology, Zhejiang University, Hangzhou, Zhejiang, China

[3] Prevention and Treatment Research Center for Senescent Disease, Zhejiang University School of Medicine, Hangzhou, Zhejiang, China

[4] Department of Gastroenterology, Second Affiliated Hospital of Zhejiang University School of Medicine, Hangzhou, Zhejiang, China

[5] Department of General Practice, Sir Run Run Shaw Hospital, Zhejiang University School of Medicine, Hangzhou, Zhejiang, China

[#] These authors contributed equally: Yadong Qi, Jiamin He, Yawen Zhang, Qiwei Ge, and Qiwen Wang.

[*] **Corresponding Authors:**

Liangjing Wang, Second Affiliated Hospital of Zhejiang University School of Medicine, 88 Jiefang Rd, Hangzhou 310009, Zhejiang, PR China, Tel +86 0571 89713905, E-mail:wangljzju@zju.edu.cn; **Shujie Chen**, Sir Run Run Shaw Hospital, Zhejiang University School of Medicine, 3 East Qingchun Rd, Hangzhou 310020, Zhejiang, PR China, Tel +86 0571 86006788, E-mail:chenshujie77@zju.edu.cn; **Jianmin Si**, Sir Run Run Shaw Hospital, Zhejiang University School of Medicine, 3 East Qingchun Rd, Hangzhou 310020, Zhejiang, PR China, Tel +86 0571 86006788, E-mail: sijm@zju.edu.cn

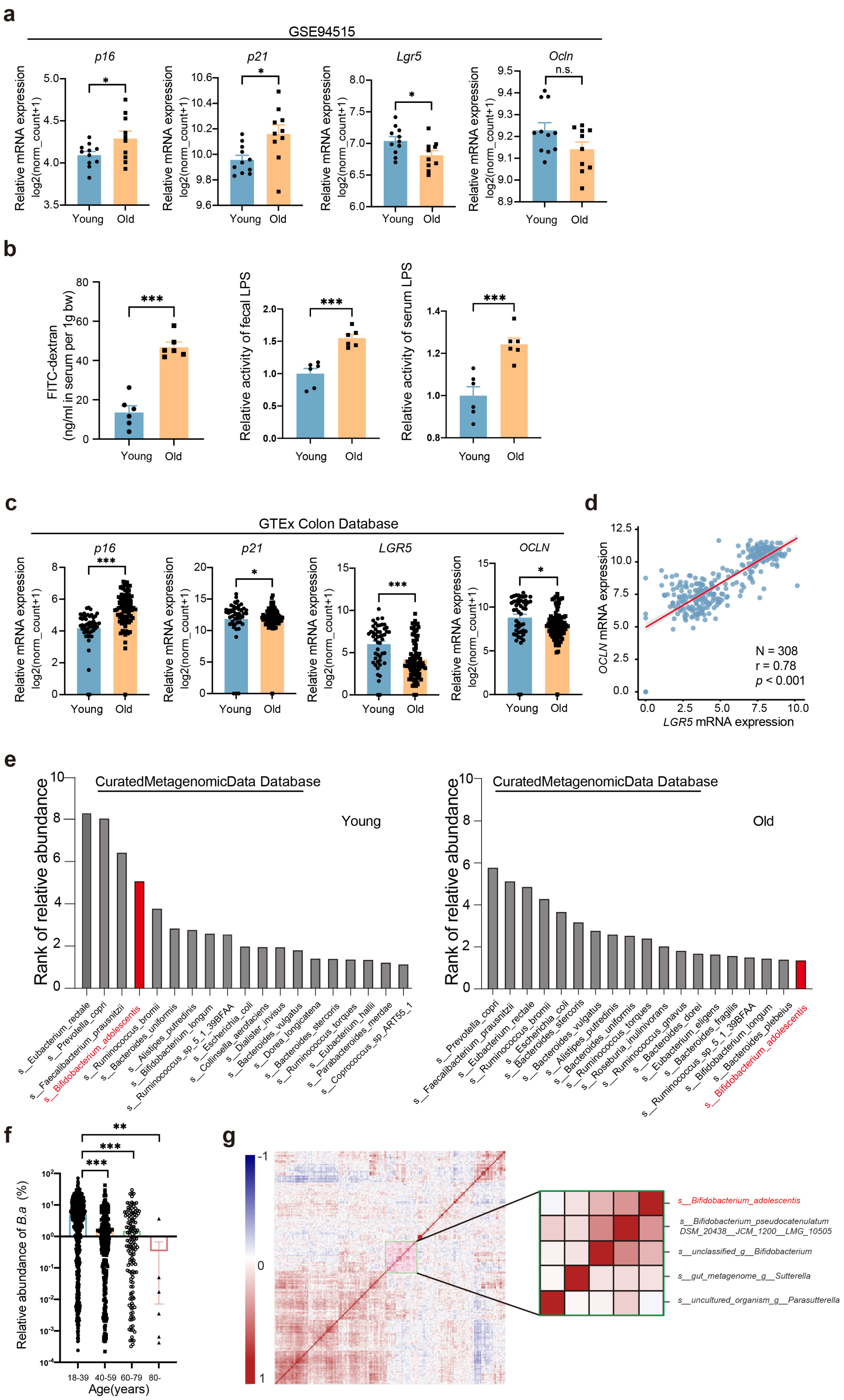

a
GSE94515
p16
p21
Lgr5
Ocln
Relative mRNA expression
log2(norm_count+1)
Young
Old
n.s.
b
FITC-dextran
(ng/ml in serum per 1g bw)
Relative activity of fecal LPS
Relative activity of serum LPS
c
GTEx Colon Database
p16
p21
LGR5
OCLN
d
OCLN mRNA expression
LGR5 mRNA expression
N = 308
r = 0.78
p < 0.001
e
CuratedMetagenomicData Database
Rank of relative abundance
Young
Old
s__Eubacterium_rectale
s__Prevotella_copri
s__Faecalibacterium_prausnitzii
s__Bifidobacterium_adolescentis
s__Ruminococcus_bromii
s__Bacteroides_uniformis
s__Alistipes_putredinis
s__Bifidobacterium_longum
s__Ruminococcus_sp_5_1_39BFAA
s__Escherichia_coli
s__Collinsella_aerofaciens
s__Dialister_invisus
s__Bacteroides_vulgatus
s__Dorea_longicatena
s__Bacteroides_stercoris
s__Ruminococcus_torques
s__Eubacterium_hallii
s__Parabacteroides_merdae
s__Coprococcus_sp_ART55_1
s__Bacteroides_stercoris
s__Roseburia_inulinivorans
s__Ruminococcus_gnavus
s__Bacteroides_dorei
s__Eubacterium_eligens
s__Bacteroides_fragilis
s__Bacteroides_plebeius
f
Relative abundance of B.a (%)
Age(years)
18-39
40-59
60-79
80-
g
s__Bifidobacterium_adolescentis
s__Bifidobacterium_pseudocatenulatum
DSM_20438__JCM_1200__LMG_10505
s__unclassified_g__Bifidobacterium
s__gut_metagenome_g__Sutterella
s__uncultured_organism_g__Parasutterella

Supplementary Fig. 1 The association between the senescent colon and age-related *B. adolescentis*.

(**a**). Relative expression of *p21*, *p16, Lgr5* and *Ocln* gene in colon tissues of young (3 months, n=11) and old (19 months, n =10) mice (GSE94515 dataset). Data were represented as mean ± SEM. Comparisons were performed by unpaired, two-tailed *t*-test (**b**). FITC-dextran concentration in the serum in young (3-month-old) and old (12-month-old) mice group. Relative LPS levels in the fecal and serum samples in young and old mice group, n = 6 animals *per* group. Data were represented as mean ± SEM. Comparisons were performed by unpaired, two-tailed *t*-test. (**c**). Relative mRNA levels of *p21*, *p16,* LGR5 and *OCLN* genes in normal colon tissues of young (20-39 years, n = 49 individuals) and old (60-79 years, n = 90 individuals) groups from the GTEx database. Data were the mean ± SEM. Comparisons were performed by unpaired, two-tailed *t*-test (**d**). The correlation analysis of the expression of *OCLN* and *LGR5* in human colonic tissues from the GTEx database, n = 308 individuals, Spearman r = 0.78. Comparisons were performed by Spearman's correlation analysis. (**e**). Rank of relative abundance for major bacteria species in young or old populations shared at the CuratedMetagenomicData Database. The ranking order of *B. adolescentis* was marked with red. (**f**). Relative abundance of *B. adolescentis* in age subgroups in CuratedMetagenomicData. Relative abundance was represented in plots as mean ± SEM. Comparisons were performed by Kruskal-Wallis test followed by Dunn's multiple comparisons test (**g**). Co-expression relationship network of distinct differences in the microbiome composition in clinical age cohort. *$p < 0.05$, **$p < 0.01$, ***$p < 0.001$, n.s. not significant. Source data and exact *p*-value are provided as a Source Data file.

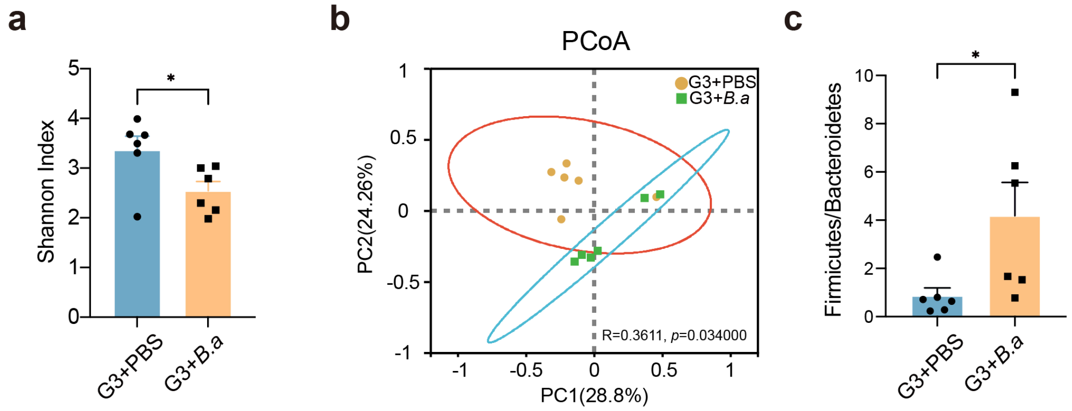

Supplementary Fig. 2. *B. adolescentis* supplementation reshaped the microbial community. (**a-b**). Theα-diversity and the β-diversity of fecal microbiomes. Data were represented as mean ± SEM. n = 6 animals *per* group. Comparisons were performed by unpaired, two-tailed *t*-test. (**c**). Proportions of the Firmicutes and the Bacteroidetes (F/B). n = 6 animals *per* group. Data were represented as mean ± SEM. Comparisons were performed by unpaired, two-tailed *t*-test.**p* < 0.05. Source data and exact *p*-value are provided as a Source Data file.

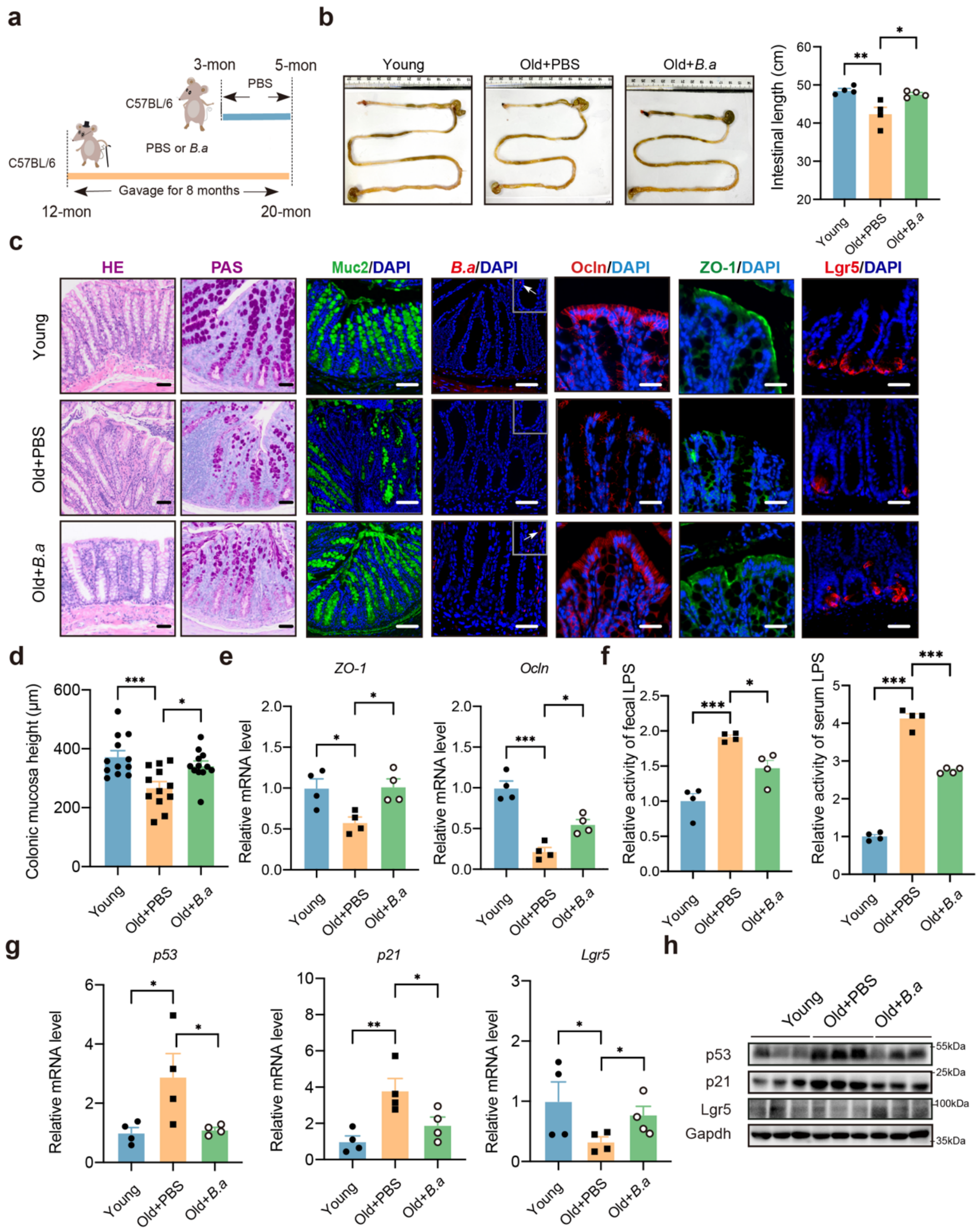

Supplementary Fig. 3. Heated-inactivated *B. adolescentis* alleviated colon senescence phenotype in natural aging mice.

(**a**). The schematic diagram of the experimental procedure in the young (3-month-old) and old (20-month-old) C57BL/6 mice. (**b**). Representative image of gross morphology and length analysis of the mouse colon. Data were mean ± SEM. n = 4 animals *per* group. Comparisons were performed by One-way ANOVA analysis followed by Tukey's multiple comparisons test. (**c**). Representative image of H&E staining and PAS staining, immunofluorescence image of Muc2, ZO-1 and Ocln and

Lgr5, FISH probe of *B. adolescentis* in the colon from Young, Old+PBS and Old+*B.a* mice. Scale bar, 50 µm. n = 6 animals *per* group. (**d**). The mucosal height was measured. n = 12 random fields per group. Data were represented as mean ± SEM. Comparisons were performed by One-way ANOVA analysis followed by Tukey's multiple comparisons test (**e**). Relative mRNA levels of *ZO-1* and *Ocln* genes in Young, Old+PBS and Old+*B.a* mice. n = 4 animals *per* group. Data were represented as mean ± SEM. Comparisons were performed by One-way ANOVA analysis followed by Tukey's multiple comparisons test. (**f**). Relative LPS levels in the fecal and serum samples in Young, Old+PBS and Old+*B.a* mice. n = 4 animals *per* group. Data were represented as mean ± SEM. Comparisons were performed by One-way ANOVA analysis followed by Tukey's multiple comparisons test. (**g**). Relative mRNA levels of *p53*, *p21* and *Lgr5* gene in Young, Old+PBS and Old+*B.a* mice. n = 4 animals *per* group. Data were represented as mean ± SEM. Comparisons in *p53*, *p21* were performed by One-way ANOVA analysis followed by Two-stage linear step-up procedure of Benjamini, Krieger and Yekutieli and in *Lgr5* were performed by Kruskal-Wallis test followed by Two-stage linear step-up procedure of Benjamini, Krieger and Yekutieli. (**h**). The protein level of p53, p21 and Lgr5 were detected by immunoblot from Young, Old+PBS and Old+*B.a* mice. n = 3 animals *per* group. *$p < 0.05$, **$p < 0.01$, ***$p < 0.001$. Source data and exact *p*-value are provided as a Source Data file.

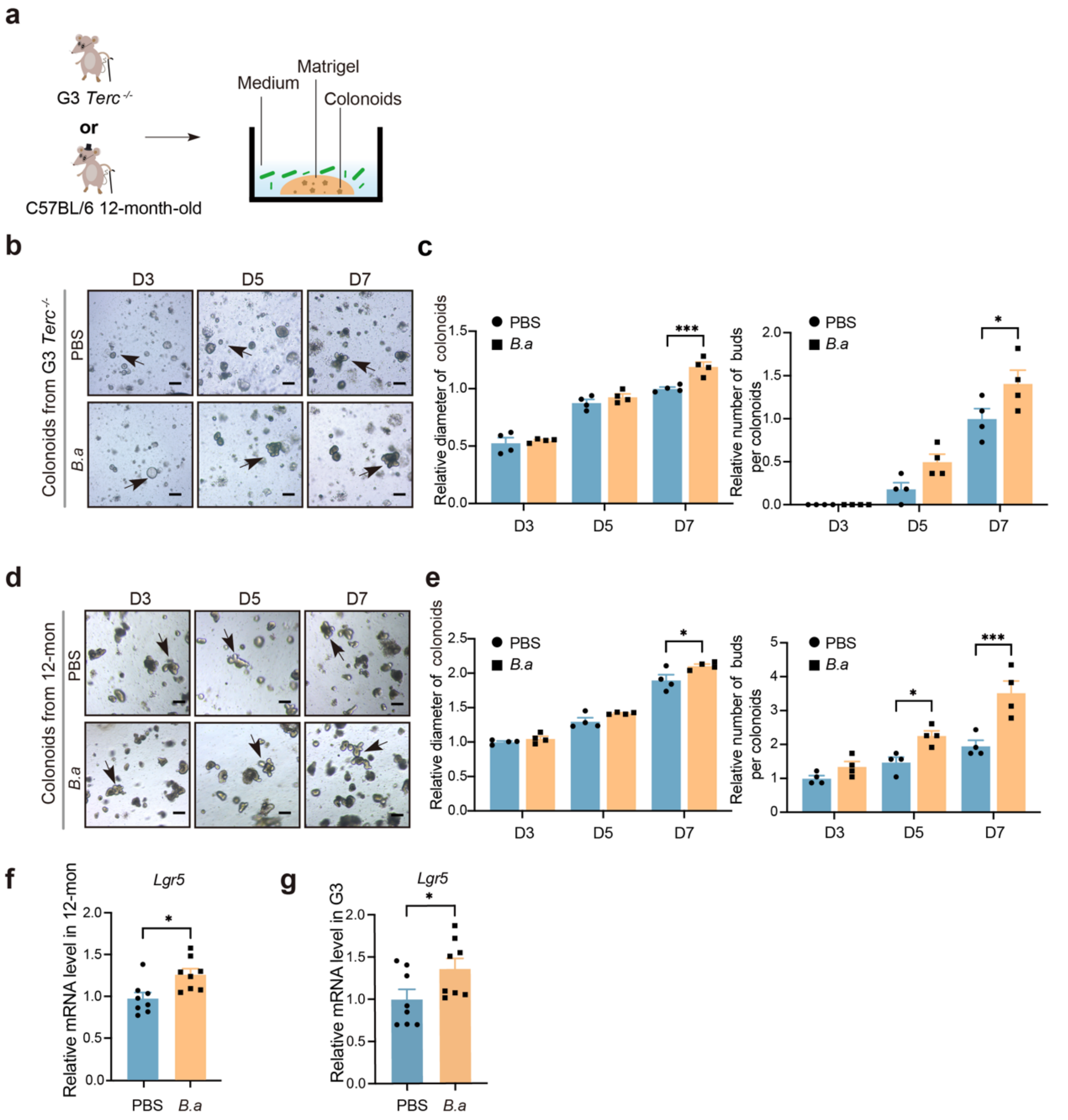

Supplementary Fig. 4. Heated-inactivated *B. adolescentis* supplementation improved the proliferation of derived colonoids from G3 *Terc*$^{-/-}$ mice and natural aging mice.

(**a**). The schematic diagram of the experimental procedure in G3 *Terc*$^{-/-}$ mice and natural aging mice *in vitro* model. (**b**). Representative images in organoid-forming capacity on day 3, 5 and 7 from PBS and *B.a* group derived from G3 *Terc*$^{-/-}$ mice crypts. Arrows indicate crypt domains. n = 4 biological replicates *per* group. Scale bar, 100 µm. (**c**). Relative size of colonoids quantified on day 3,5 and 7 and represented relative to PBS treated control. n = 4 biological replicates *per* group. Data were represented as mean ± SEM. Comparisons were performed by Two-way ANOVA analysis followed by Bonferroni's multiple comparisons test. (**d**). Representative images of organoid-forming capacity on day 3,5 and 7 from PBS and *B.a* treated group derived from 12-month mice crypts. Arrows indicate crypt domains. n = 4 biological replicates *per* group. Scale bar, 100 µm. (**e**). Relative size

of colonoids quantified on day 3, 5 and 7. n = 4 biological replicates *per* group. Data were represented as mean ± SEM. Comparisons were performed by Two-way ANOVA analysis followed by Bonferroni's multiple comparisons test. (**f-g**). Relative mRNA levels of Lgr5 gene in colonoids derived from 12-month or G3 *Terc*$^{-/-}$ mice. n = 8 biological replicates *per* group. Data were presented as the mean ± SEM. Comparisons were performed by unpaired, two-tailed *t*-test *$p < 0.05$, ***$p < 0.001$. Source data and exact *p*-value are provided as a Source Data file.

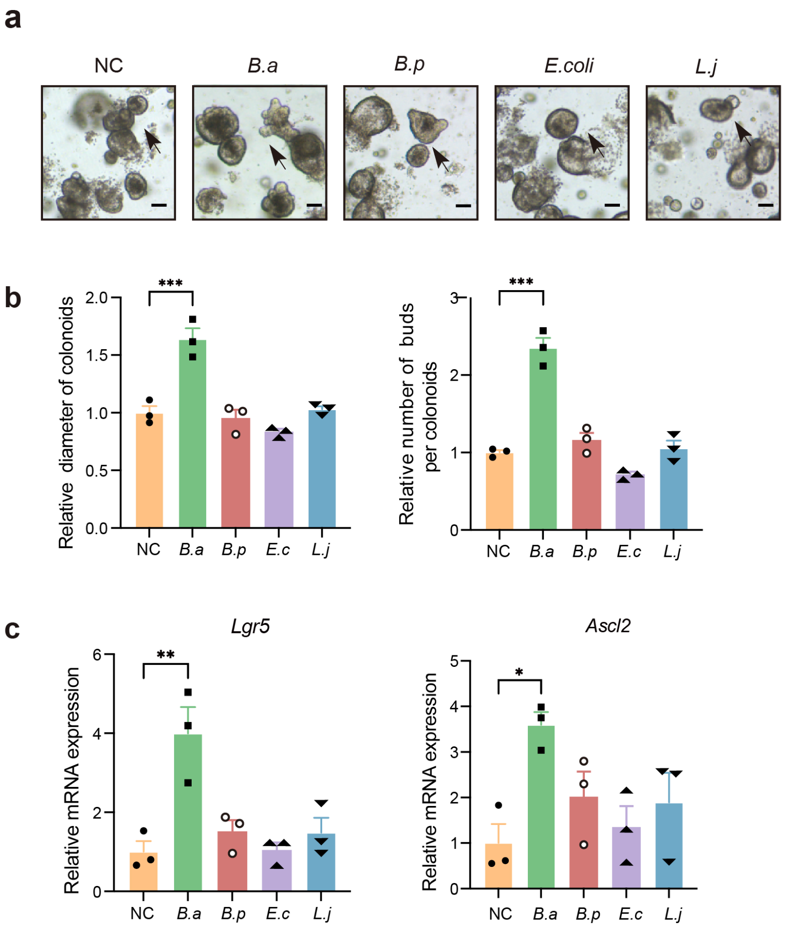

Supplementary Fig. 5. ***B. adolescentis*** **specifically improved the proliferation of derived colonoids.**

(**a-b**). Representative images in organoid-forming capacity and size of colonoids quantified from NC, *B.a*, *B.p*, *E.coli* and *L.j* group on day 7. Scale bar, 100 μm. Arrows indicate crypt domains. n = 3 independent experiments. Data were represented as mean ± SEM and represented relative to NC control. Comparisons were performed by One-way ANOVA analysis followed by followed by Tukey's test. (**c**). The expression of *Lgr5* and *Ascl2* genes were detected by qPCR from NC, *B.a*, *B.p*, *E.coli* and *L.j* group. n = 3 independent experiments. Data were represented as mean ± SEM. Comparisons were performed by One-way ANOVA analysis followed by followed by Tukey's test. *$p < 0.05$, **$p < 0.01$, ***$p < 0.001$. *L.j*, *Lactobacillus johnsonii*. *B.p*, *Bifidobacterium pseudolongum*. *E. coli*. *Escherichia coli*. *B.a*, *Bifidobacterium adolescentis*. Source data and exact *p*-value are provided as a Source Data file.

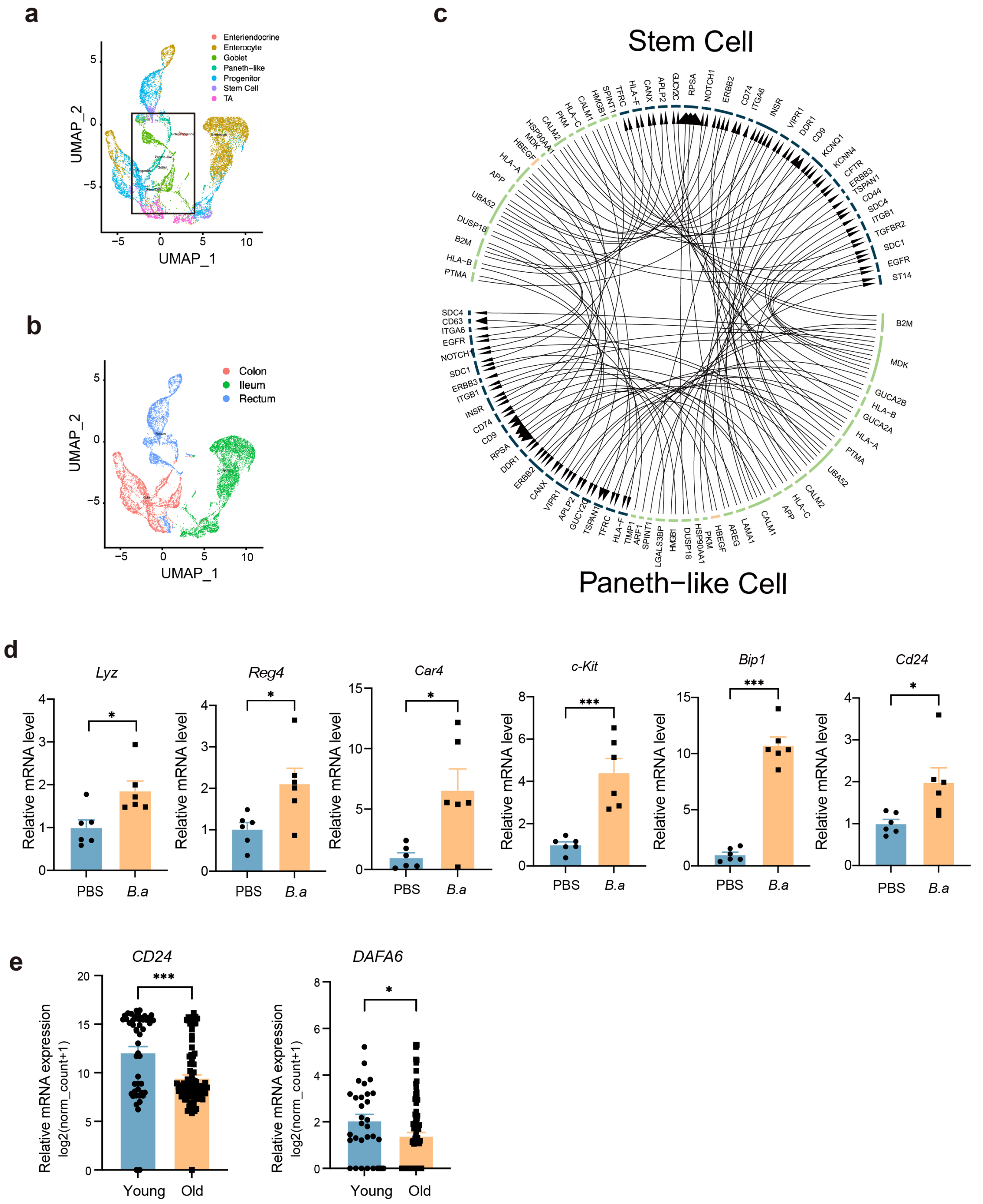

Supplementary Fig. 6. *B. adolescentis* increased Paneth-like cells development

(**a**). Different cell clusters in human gut single-cell transcriptome data (GSE125970). (**b**). Different tissue types in human gut single-cell transcriptome data (GSE125970). (**c**). Detail genes involved in cell communication between PLCs and ISCs in the single-cell transcriptome. (**d**). Relative mRNA levels of Lyz, Reg4, *Car4*, *c-Kit*, *Bip1* and *Cd24* genes in colonoid model. n = 6 biological replicates *per* group. Data were presented as the mean ± SEM. Comparisons were performed by unpaired, two-tailed *t*-test. (**e**). Relative expression of *CD24* and *DAFA6* in normal colon tissue of GTEx database. Data were represented as mean ± SEM. Comparisons were performed by unpaired, two-

tailed *t*-test in *CD24* and Mann-Whitney test in *DAFA6*. *$p < 0.05$, ***$p < 0.001$. Source data and exact *p*-value are provided as a Source Data file.

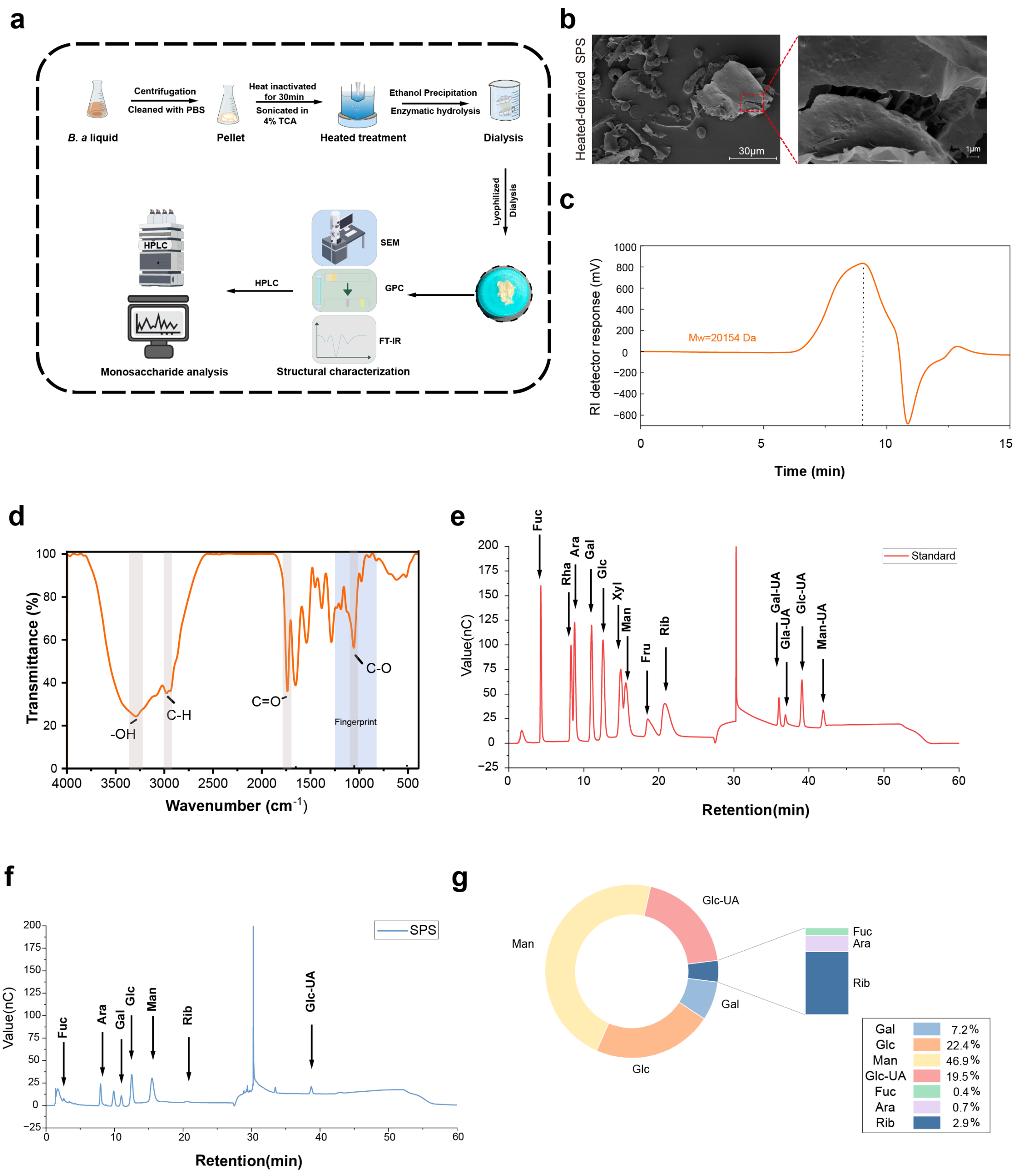

Supplementary Fig. 7. Structural characterization and monosaccharide composition analysis of SPS

(**a**). SPS extraction diagrams from heated-inactivated *B. adolescentis* (95 °C). (**b**). Scanning electron microscopy (SEM) images (at 500× and 5000× magnification) of heated-derived SPS. (**c**). Gel permeation chromatography (GPC) profiles of heated-derived SPS, the average molecular weight (Mw) was calculated. (**d**). Fourier-transform infrared spectroscopy (FT-IR) of heated-derived SPS, labeling the characteristic functional groups of the polysaccharide based on chemical bonds (3307 cm^{-1}, 2940 cm^{-1}, 1741 cm^{-1} and 1056 cm^{-1} represents O-H stretching, C-H stretching C=O) stretching and C-OH stretching vibrations, respectively), the gray shaded region corresponds to the fingerprint

spectrum of the polysaccharide. (**e**). Chromatogram profiles of the monosaccharide. Fuc, Fucose; Rha, Rhamnose; Ara, Arabinose; Gal, Galactose; Glc, Glucose; Xyl, Xylose; Man, Mannose; Fru, Fructose; Rib, Ribose; Gal-UA, Galacturonic Acid; Glc-UA, Glucuronic Acid; Man-UA, Mannuronic Acid; Gul-UA, Guluronic Acid. (**f**). Chromatogram profiles of the heated-derived SPS, labeling the monosaccharide based on profiles of the standard monosaccharide. (**g**). The monosaccharide composition of the heated-derived SPS, the number expressed as the mass percentage of SPS. SPS, Soluble polysaccharides. Source data and exact *p*-value are provided as a Source Data file.

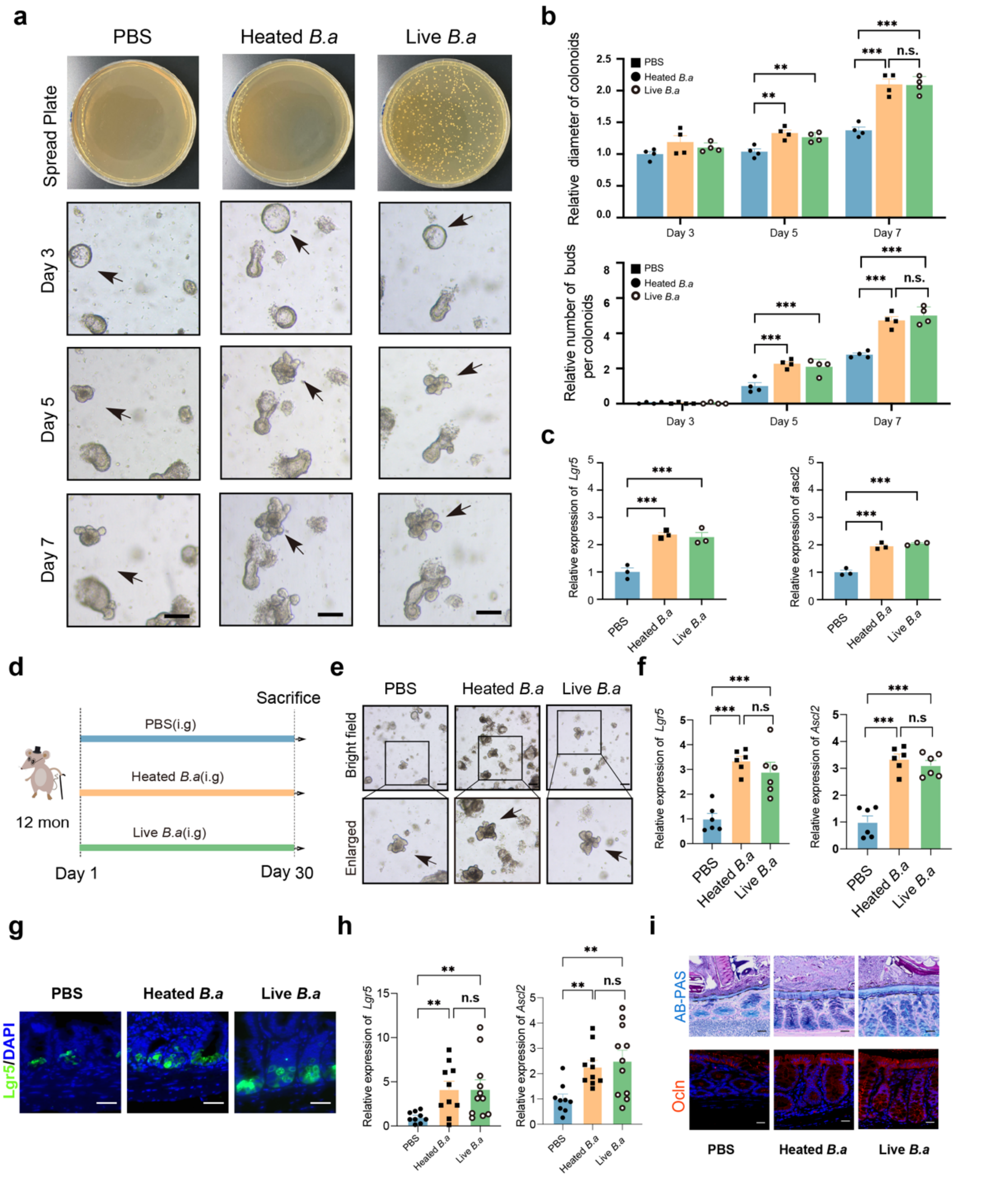

Supplementary Fig. 8. Live *B. adolescentis* demonstrated a comparable effect as heat-inactivated *B. adolescentis in vitro* colonoids

(**a**). Representative images are shown in the Dilution plating (above) and organoid-forming capacity of crypts (below) from PBS, heat-inactivated *B. adolescentis* and live *B. adolescentis* treated group. Arrows indicate crypt domains. Scale bar, 100 μm. n = 4 independent experiments. (**b**). The relative size of colonoids was quantified on days 3, 5 and 7 and represented relative to the PBS-treated group. n = 4 independent experiments. Data were represented as mean ± SEM. Comparisons were performed by Two-way ANOVA analysis followed by Tukey's test. (**c**). Relative expression of Lgr5

and Ascl2 gene in PBS, heat-inactivated *B. adolescentis* and live *B. adolescentis* treated group. n =3 independent experiments. Data were represented as mean ± SEM. Comparisons were performed by One-way ANOVA analysis followed by Tukey's test. (**d**). The schematic diagram of the 12-month mice experimental procedure in PBS group (n=9), heat-inactivated *B. adolescentis* group (n=10) and live *B. adolescentis* group (n=11). (**e**).Representative images in the organoid-forming capacity of crypts (Right) from PBS group, heat-inactivated *B. adolescentis* group and live *B. adolescentis* group. Arrows indicate crypt domains. Scale bar, 100 μm. n = 6 mice *per* group. (**f**) .Relative expression of Lgr5 and Ascl2 gene in colonoids derived from PBS, heat-inactivated *B. adolescentis* and live *B. adolescentis* treated group. n = 6 mice *per* group. Comparisons were performed by One-way ANOVA analysis followed by Tukey's test. (**g**). Representative image of *Lgr5* genes was detected in colon tissue from PBS group (n=9), heat-inactivated *B. adolescentis* group (n=10) and live *B. adolescentis* group (n=11). (**h**).Relative expression of Lgr5 and Ascl2 gene in colon tissue from PBS (n=9), heat-inactivated *B. adolescentis* (n=10) and live *B. adolescentis* treated group(n=11). Data were represented as mean ± SEM. Comparisons were performed by One-way ANOVA analysis followed by Tukey's test. (**i**). Representative image of AB-PAS staining and immunofluorescence image of Ocln in the colon from PBS group (n=9), heat-inactivated *B. adolescentis* group (n=10) and live *B. adolescentis* group (n=11). Scale bar, 50 μm. **$p < 0.01$, ***$p < 0.001$, n.s. not significant. Source data and exact *p*-value are provided as a Source Data file.

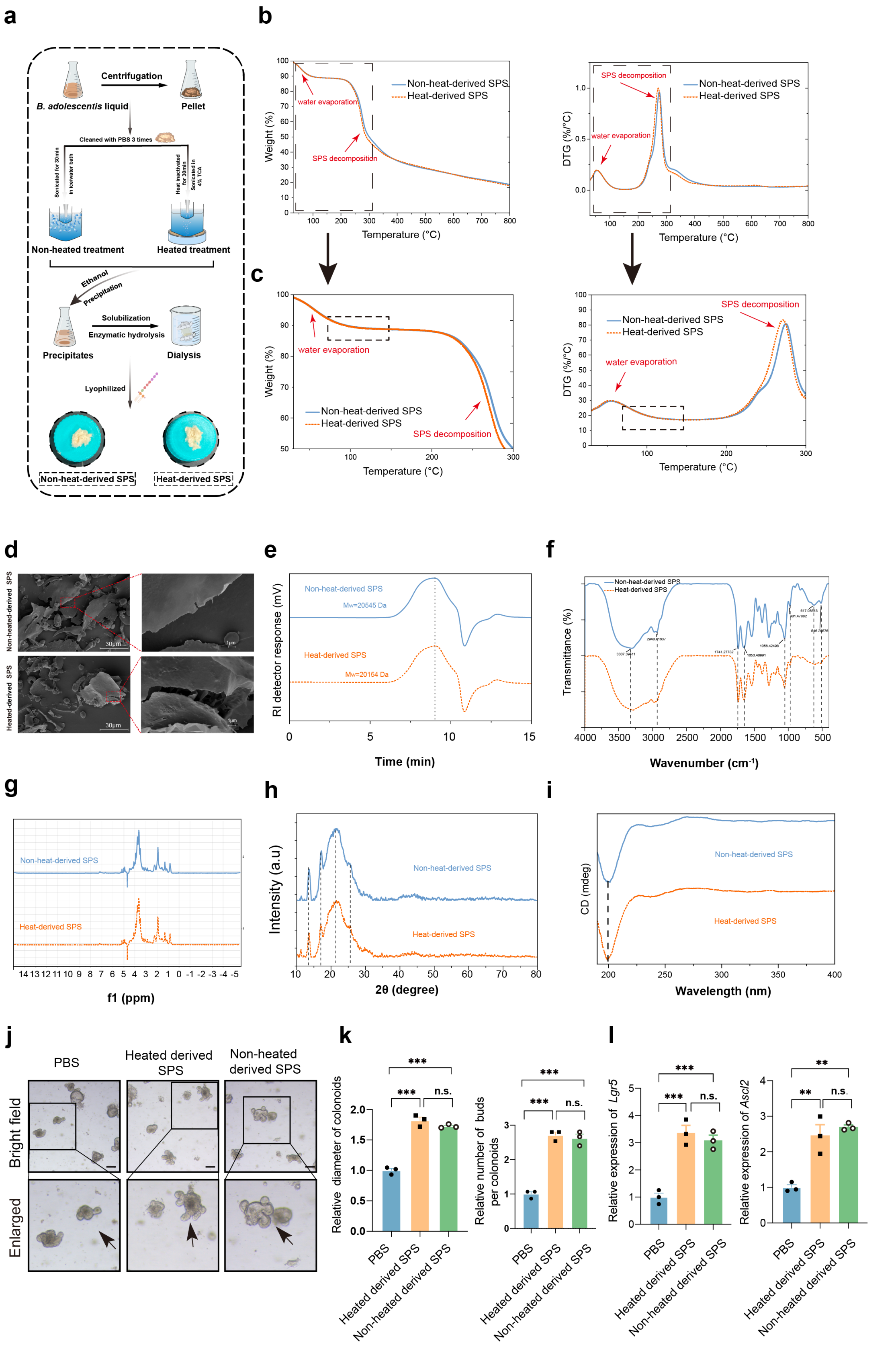

Supplementary Fig. 9 The soluble polysaccharides (SPS) derived from both live *B. adolescentis* and heat-inactivated *B. adolescentis* demonstrated similar structural characterization and comparable promotion effects *in vitro* colonoids models.

(**a**). Schematic diagrams of extracted SPS from heated-inactivated (95 °C) and non-heated *B. adolescentis*. (**b-c**). Thermal gravimetric analysis (TGA) and Derivative Thermogravimetry (DTG) curves of non-heated-derived SPS and heated-derived SPS (Above). Locally magnified curves at around 100 °C (Below). (**d**). Scanning electron microscopy (SEM) images (at 500 × and 5000 × magnification) of non-heated-derived SPS and heated-derived SPS. (**e**). Gel permeation chromatography (GPC) profiles of non-heated-derived SPS and heated-derived SPS. (**f**). Fourier-transform infrared spectroscopy (FT-IR) of non-heated-derived SPS and heated-derived SPS. (**g**).Nuclear ^{1}H magnetic resonance (^{1}H-NMR) spectrum of non-heated-derived SPS and heated-derived SPS. (**h**). X-ray diffraction (XRD) patterns of non-heated-derived SPS and heated-derived SPS. (**i**). Circular dichroism (CD) spectrum of non-heated-derived SPS and heated-derived SPS.(**j**). Representative images in the organoid-forming capacity of crypts from PBS, heated derived SPS, non-heated derived SPS treated group. n = 3 independent experiments. Arrows indicate crypt domains. Scale bar, 100 µm. (**k**). Relative size of colonoids quantified on 7 and represented relative to PBS treated group. n = 3 independent experiments. Data were represented as mean ± SEM. Comparisons were performed by One-way ANOVA analysis followed by Tukey's multiple comparisons test. (**l**). Relative expression of *Lgr5* and Ascl2 gene in PBS, heated derived SPS, non-heated derived SPS treated group. n = 3 independent experiments. Data were represented as mean ± SEM. Comparisons were performed by One-way ANOVA analysis followed by Tukey's multiple comparisons test, $**p < 0.01$, $***p < 0.001$. n.s. not significant. SPS, Soluble polysaccharides. Source data and exact *p*-value are provided as a Source Data file.

Supplementary Table 1: Primers used for validation of the gene expression

Primers	Sequencing(5′-3′)
universal Eubacteria 16s-F	*CGGCAACGAGCGCAACCC*
universal Eubacteria 16s-R	*CCATTGTAGCACGTGTGTAGCC*
B. adolescentis-F	*CTCCGCCGCTGATCCGGAAGTCG*
B. adolescentis-R	*AACCAACTCGGCGATGTGGACGACA*
mouse-*p16*-F	*TGTTGAGGCTAGAGAGGATCTTG*
mouse-*p16*-R	*CGAATCTGCACCGTAGTTGAGC*
mouse-*p21*-F	*TCGCTGTCTTGCACTCTGGTGT*
mouse-*p21*-R	*CCAATCTGCGCTTGGAGTGATAG*
mouse-*p53*-F	*CTGGTTAGTCCTGAGACAGAGG*
mouse-*p53*-R	*AGATGCAGCCAAACACAGGCAC*
mouse-*Lgr5*-F	*CCTACTCGAAGACTTACCCAGT*
mouse-*Lgr5*-R	*GCATTGGGGTGAATGATAGCA*
mouse-*c-Myc*-F	*GTCTTTCCCTACCCGCTCAA*
mouse-c-*Myc*-R	*TCTTCTTGCTCTTCTTCAGAGTCG*
mouse-*CyclinD1*-F	*GCAGAAGGAGATTGTGCCATCC*
mouse-*CyclinD1*-R	*AGGAAGCGGTCCAGGTAGTTCA*
mouse-*Axin2*-F	*ATGGAGTCCCTCCTTACCGCAT*
mouse-*Axin2*-R	*GTTCCACAGGCGTCATCTCCTT*
mouse-*Notch1*-F	*CACCAGGGTGGTCAGGAAAA*
mouse-*Notch1*-R	*GGGCAGCGACAGATGTATGA*
mouse-*Dll1*-F	*GCAGGACCTTCTTTCGCGTAT*
mouse-*Dll1*-R	*AAGGGGAATCGGATGGGGTT*
mouse-*Dll4*-F	*TTCCAGGCAACCTTCTCCGA*
mouse-*Dll4*-R	*ACTGCCGCTATTCTTGTCCC*
mouse-*Jagged1*-F	*CCTCGGGTCAGTTTGAGCTG*
mouse-*Jagged1*-R	*CCTTGAGGCACACTTTGAAGTA*
mouse-*Wnt3a*-F	*CTCGCTGGCTACCCAATTTG*
mouse-*Wnt3a*-R	*CTTCACACCTTCTGCTACGCT*
mouse-*Wnt3*-F	*CAAGCACAACAATGAAGCAGGC*
mouse-*Wnt3*-R	*TCGGGACTCACGGTGTTTCTC*
mouse-*Wnt1*-F	*ATGAACCTTCACAACAACGAG*
mouse-*Wnt1*-R	*GGTTGCTGCCTCGGTTG*
mouse-*Wnt5a*-R	*GGAACGAATCCACGCTAAGGGT*
mouse-*Wnt5a*-F	*AGCACGTCTTGAGGCTACAGGA*
mouse-*Wnt11*-F	*GCCTGTGAAGGACTCAGAACTTG*
mouse-*Wnt11*-R	*AGCTGTCACTGCCGTTGGAAGT*
mouse-*Lyz1*-F	*TACAACCGTGGAGACCGAAGCA*
mouse-*Lyz1*-R	*TGGCTGCAGTGATGTCATCCTG*
mouse-R*eg4*-F	*CTGGCTATCAGAGAAACCTGCC*
mouse-R*eg4*-R	*CTGGCTTCACTCTTTGTCCTGG*
mouse-*Car4*-F	*CCCTCTACTGAAGACTCAGGC*
mouse-*Car4*-R	*TCTCCTCCGATAATGCACGC*
mouse-*cKit*-F	*TCATCGAGTGTGATGGGAAA*
mouse-*cKit*-R	*GGTGACTTGTTTCAGGCACA*
mouse-*Cd24a*-F	*CCAAGCCTGTCCCGTTCC*

mouse-*Cd24a*-R	*GGTTGCAGTAAATCTGCGTGG*
mouse-*Spib*-F	*TGCTCTGAACCACCATGCTT*
mouse-*Spib*-R	*CCCATGTAGAGTCAAGGCCC*
mouse-*β-Actin*-F	*TGTTACCAACTGGGACGACA*
mouse-*β-Actin*-R	*GGGGTGTTGAAGGTCTCAAA*
human-*LGR5_1F*	*CCGCTTCCTGGAGGAGTTAC*
human-*LGR5_1R*	*GCATCCAGACGCAGGGATTG*
human-*LYZ_3F*	*ACTACAATGCTGGAGACAGAAGC*
human-*LYZ_3R*	*GCACAAGCTACAGCATCAGCGA*
human-*REG4_3F*	*TGAGGAACTGGTCTGATGCCGA*
human-*REG4_3R*	*TCCATATCGGCTGGCTTCTCTG*
human-*β-ACTIN*-F	*GTGAAGGTGACAGCAGTCGGTT*
human-*β-ACTIN*-R	*GAAGTGGGGTGGCTTTTAGGA*

Supplementary Table S2: The gender and age of participants in human samples

Sample ID	Gender	Age
QN1	M	39
QN2	F	44
QN3	M	39
QN4	F	45
QN5	F	65
QN6	M	45
QN7	M	55
QN8	M	53
QN9	F	45
QN10	M	43
QN11	F	40
QN13	M	67
QN14	F	54
QN15	M	62
QN16	M	40
QN18	F	47
QN19	F	61
QN20	M	69
QN21	F	67
QN23	F	62
QN24	F	45
QN26	M	44
N1	F	52
N2	M	58
N3	M	50
N4	F	62
N5	M	59
N6	M	63
N7	M	39
N8	F	72
N9	F	73
N10	F	71
N11	M	49
N12	F	49
N13	F	69
N14	F	70
N20	F	43
N21	F	32
N22	M	54
N23	F	65
