# Supplementary material for: Heat-inactivated Bifidobacterium adolescentis ameliorates colon senescence through Paneth-like-cell-mediated stem cell activation
Source: Nat Commun. 2023 Sep 30;14:6121. doi: 10.1038/s41467-023-41827-0 (PMC10542354; doi:10.1038/s41467-023-41827-0)
Supplement: Supplementary file 2 — Reporting Summary [file 41467_2023_41827_MOESM2_ESM.pdf]

nature portfolio

Corresponding author(s): Liangjing Wang

Last updated by author(s): Sep 1, 2023

Reporting Summary

Nature Portfolio wishes to improve the reproducibility of the work that we publish. This form provides structure for consistency and transparency in reporting. For further information on Nature Portfolio policies, see our Editorial Policies and the Editorial Policy Checklist.

Statistics

For all statistical analyses, confirm that the following items are present in the figure legend, table legend, main text, or Methods section.

n/a	Confirmed	
☐	☒	The exact sample size (*n*) for each experimental group/condition, given as a discrete number and unit of measurement
☐	☒	A statement on whether measurements were taken from distinct samples or whether the same sample was measured repeatedly
☐	☒	The statistical test(s) used AND whether they are one- or two-sided<br>*Only common tests should be described solely by name; describe more complex techniques in the Methods section.*
☒	☐	A description of all covariates tested
☐	☒	A description of any assumptions or corrections, such as tests of normality and adjustment for multiple comparisons
☐	☒	A full description of the statistical parameters including central tendency (e.g. means) or other basic estimates (e.g. regression coefficient) AND variation (e.g. standard deviation) or associated estimates of uncertainty (e.g. confidence intervals)
☐	☒	For null hypothesis testing, the test statistic (e.g. *F*, *t*, *r*) with confidence intervals, effect sizes, degrees of freedom and *P* value noted<br>*Give P values as exact values whenever suitable.*
☒	☐	For Bayesian analysis, information on the choice of priors and Markov chain Monte Carlo settings
☒	☐	For hierarchical and complex designs, identification of the appropriate level for tests and full reporting of outcomes
☐	☒	Estimates of effect sizes (e.g. Cohen's *d*, Pearson's *r*), indicating how they were calculated

Our web collection on statistics for biologists contains articles on many of the points above.

Software and code

Policy information about availability of computer code

Data collection	Commercia softwares equipped by ROCHE LightCycler®480 System (Rotor gene 6000 Software, Sydney, Australia);Inverted microscope (ZEISS LSM 800, Munich, Germany);Zen image program(ZEISS LSM 800, Munich, Germany) ;ChemiDoc Touch Imaging System(Bio-Rad);ICS5000+ (Thermo Scientific);GX FT-IR system (PerkinElmer, USA);Gel-permeation chromatography (GPC) ;scanning electron microscope (Nova Nano 450., Thermo FEI).
Data analysis	Image Lab Software v6.1 (Bio Rad) for Immunoblots; QuPath (Edinburgh, version:0.3.0) and Fiji(NIH, version:1.2.0) for Raw images;GraphPad Prism (GraphPad Software 6.0.0, San Diego, CA, USA) and SPSS software version 19.0 (SPSS, Inc., Chicago, Illinois) for plot and analysis;R (version 3.6.3,https://www.r-project.org) and RStudio (version 1.3.1093, https://www.rstudio.com) software for microbiome data;chromeleon 7.2 CDS (Thermo Scientific); 16S rRNA gene sequence data and single-cell transcriptome data analyses were performed using standard QIIME2 (2017.6) (https://qiime2.org/) and Seurat (4.3.0) (https://satijalab.org/seurat/) analysis pipelines. Analysis R scripts employing R packages are available from https://github.com/Yadong-Qi/Paneth-like-cells-AND-stem-cell-activation.

For manuscripts utilizing custom algorithms or software that are central to the research but not yet described in published literature, software must be made available to editors and reviewers. We strongly encourage code deposition in a community repository (e.g. GitHub). See the Nature Portfolio guidelines for submitting code & software for further information.

Data

Policy information about availability of data

All manuscripts must include a data availability statement. This statement should provide the following information, where applicable:

- Accession codes, unique identifiers, or web links for publicly available datasets
- A description of any restrictions on data availability
- For clinical datasets or third party data, please ensure that the statement adheres to our policy

The raw 16S rRNA gene sequences data generated in this study have been deposited in the Genome Sequence Archive (GSA) database, under accession code CRA008671 [https://ngdc.cncb.ac.cn/search/?dbId=gsa&q=CRA008671]. The Spatial transcriptomics and single-cell transcriptome data used in this study are available in the Gene Expression Omnibus (GEO) database under accession code GSE125970 [https://www.ncbi.nlm.nih.gov/geo/query/acc.cgi?acc=GSE125970]. The transcriptome of mice colonic tissues data used in this study are available in the Gene Expression Omnibus (GEO) database under accession code GSE94515 [https://www.ncbi.nlm.nih.gov/geo/query/acc.cgi?acc= GSE94515]. GMrepo database is accessible by link (https://gmrepo.humangut.info/), CuratedMetagenomicData database is accessible by link (https://waldronlab.io/curatedMetagenomicData/), GTEx database coubler obtain from the GTEx portal website (http://www.gtexportal.org).Other data from the findings of this study are available from the corresponding author upon request. Source data are provided in this paper.

Research involving human participants, their data, or biological material

Policy information about studies with human participants or human data. See also policy information about sex, gender (identity/presentation), and sexual orientation and race, ethnicity and racism.

Reporting on sex and gender	Sex and gender Information about Microbiota online data(GMrepo database /CuratedMetagenomicData database) are available in website(https://gmrepo.humangut.info/ AND doi:10.18129/B9.bioc.curatedMetagenomicData).The part of this study involving healthy people did not design for gender or sex.
Reporting on race, ethnicity, or other socially relevant groupings	Study not use the constructs of race and/or ethnicity.Race/ethnicity not be used as proxies for other variables,
Population characteristics	Information about Microbiota online data(GMrepo database /CuratedMetagenomicData database) are available in website(https://gmrepo.humangut.info/ AND doi:10.18129/B9.bioc.curatedMetagenomicData).<br>For human colonic tissue samples,covariate-relevant population characteristics of the human research participants (e.g. age(32-73ys), gender(22F:18M)) is provied in Supplementary Table S2.
Recruitment	Recruitment at Sir Run Run Shaw Hospital of Zhejiang University School of Medicine, we confirm that human colonic samples were obtained as routine procedure.
Ethics oversight	All participants provided written informed consent before collection and the Clinical Research Ethics Committee of the Sir Run Run Shaw Hospital of Zhejiang University School of Medicine approved the study protocol (20211103-35).

Note that full information on the approval of the study protocol must also be provided in the manuscript.

Field-specific reporting

Please select the one below that is the best fit for your research. If you are not sure, read the appropriate sections before making your selection.

☒ Life sciences ☐ Behavioural & social sciences ☐ Ecological, evolutionary & environmental sciences

For a reference copy of the document with all sections, see nature.com/documents/nr-reporting-summary-flat.pdf

Life sciences study design

All studies must disclose on these points even when the disclosure is negative.

Sample size	No statistical method was used to predetermine sample size.<br>For in vitro experiments such as Western blot, qPCR at least three biological replicateswere used per group for minimal statistics requirements.<br>For in vivo studies, the sample size was determined to be sufficient to obtain the statistical difference between groups, each experiment was performed at least three samples.<br>For human samples, samples were collected until the sample size was sufficient to give reliable estimates.
Data exclusions	There are no data exclusions.
Replication	All experiments of this study have been replicated multiple times(n>3) and corroborated by several models. All the western blot, qPCR were carried out at least three independent times with the same results.

Randomization	Samples and organisms were randomly allocated to experimental groups. Mice were age matched.
Blinding	For cell and biochemical experiments,no specific blinding was applied since they were assigned into groups including relevant controls and analysis was done objectively and without bias.<br>For the animal assays, the investigators were blinded to group allocation during experiments .

Reporting for specific materials, systems and methods

We require information from authors about some types of materials, experimental systems and methods used in many studies. Here, indicate whether each material, system or method listed is relevant to your study. If you are not sure if a list item applies to your research, read the appropriate section before selecting a response.

Materials & experimental systems

n/a	Involved in the study	
☐	☒	Antibodies
☐	☒	Eukaryotic cell lines
☒	☐	Palaeontology and archaeology
☐	☒	Animals and other organisms
☒	☐	Clinical data
☒	☐	Dual use research of concern
☒	☐	Plants

Methods

n/a	Involved in the study	
☒	☐	ChIP-seq
☒	☐	Flow cytometry
☒	☐	MRI-based neuroimaging

Antibodies

Antibodies used	Anti-Muc2 antibody (Servicebio, GB14110, 1:500);<br>anti-LGR5 (OriGene, TA503316,1:100;<br>anti-active β-Catenin(CST, #8814,1:100<br>anti-Lysozyme(Abcam, ab108508,1:100)<br>anti-p53 (ab167161, Abcam,1:1000),<br>anti-p21 (sc-6246,Santa Cruz,1:500),<br>anti-Lgr5 (Bioss, bs-20747R,1:1000),<br>anti-c-Myc (CST, #5605S,1:1000),<br>anti-Cyclin D1 (CST, # 2978, 1:1000),<br>anti-active β-Catenin (CST. # 8814, 1:1000),<br>anti-Reg4 (Abclonal, A13129, 1:1000),<br>anti-β-actin (ET1701-80, HUABIO,1:1000),<br>anti-Gapdh (ET1601-4, HUABIO,1:1000) ;
Validation	Protein are identified based on molecular weight (kDa).

Eukaryotic cell lines

Policy information about cell lines and Sex and Gender in Research

Cell line source(s)	The HEK-Blue™ hTLR4 cells were kindly provided by Prof. Yiqi Wang (Zhejiang Chinese Medical University, China)
Authentication	None of the cell lines used were authenticated
Mycoplasma contamination	All cell lines used in this study were tested negative for mycoplasma contamination.
Commonly misidentified lines<br>(See ICLAC register)	There is no commonly misidentified cell line used in this study.

Animals and other research organisms

Policy information about studies involving animals; ARRIVE guidelines recommended for reporting animal research, and Sex and Gender in Research

Laboratory animals	Heterozygous telomerase RNA component Terc knockout mice (G0 Terc+/-) in the C57BL/6 background were gifted by Dr. Song. Generation one knockout animals (G1 Terc -/-) were derived from G0 Terc+/- mice and finally generate G3 Terc-/- for experiments 6-8-week-old wild-type (WT+PBS, n = 11 and 8 males, 3 females) littermates were administrated intragastrically with PBS as the control group. 6-8 week-old Terc-/- G3 littermates were randomly assigned to the PBS group (G3+PBS, n = 9 and 5 males, 4 females) and B. adolescentis group (G3+B.a, n = 12 and 7 males, 5 females) administrated intragastrically with heat-inactivated B. adolescentis until natural death or sacrifice at 7 months old. For other animal experiments, C57BL/6 mice (3-month-old and 12-month-old, male) were used in the experiment. the number of each experiment is listed in the figure legends. All mice were maintained at a specific pathogen-free (SPF) level animal facility in Sir Run Run Shaw Hospital with a room temperature of 20-22? and humidity (45-65°C).

	Mice were provided with a standard laboratory diet and water freely in ventilated cages with a 12-hour light/12-hour dark circadian cycle. All animal studies were performed in accordance with the guidelines of the Institutional Animal Use and the Animal Experimentation Ethics Committee at Zhejiang University<br>For other animal experiments, C57BL/6 mice (3-month-old and 12-month-old, male) were used in the experiment. t
Wild animals	No wild animals were used in the study.
Reporting on sex	WT 6- to 8-week-old C57BL/6 (n = 11 and 8 males, 3 females); 6- to 8-week old Terc −/− G3 (n = 9 and 5 males, 4 females) ;B. adolescentis group (n = 12 and 7 males, 5 females).For other animal experiments, C57BL/6 mice (3-month-old and 12-month-old, male) The part of this study involving healthy people did not design for gender or sex.
Field-collected samples	no field collected samples were used in the study.
Ethics oversight	All animal studies were performed in accordance with the guidelines of the Institutional Animal Use and the Animal Experimentation Ethics Committee at Zhejiang University.

Note that full information on the approval of the study protocol must also be provided in the manuscript.
